# Microorganisms in wild European reptiles: bridging gaps in neglected conditions to inform disease ecology research

**DOI:** 10.1016/j.ijppaw.2025.101113

**Published:** 2025-07-05

**Authors:** Matteo Riccardo Di Nicola, Selene Rubiola, Anna Cerullo, Andrea Basciu, Claudia Massone, Thomas Zabbia, Jean Lou CM Dorne, Pier Luigi Acutis, Daniele Marini

**Affiliations:** a Istituto Zooprofilattico Sperimentale del Piemonte, Liguria e Valle d'Aosta, Via Bologna 148, 10154, Turin, Italy; b Faculty of Veterinary Medicine, Department of Pathobiology, Pharmacology and Zoological Medicine, Wildlife Health Ghent, Ghent University, Merelbeke, 9820, Belgium; c Department of Veterinary Sciences, University of Turin, L.go Braccini 5, Grugliasco, Turin, 10095, Italy; d WAY4WARD, Via Ruggero Bonghi 11/B, 00184, Roma, Italy; e Independent Researcher, Via Romana, 06126, Perugia, Italy; f Independent Researcher, Via Ungaretti, 22030, Orsenigo, Italy; g Methodological and Scientific Support Unit, European Food Safety Authority, Via Carlo Magno 1A, 43100, Parma, Italy; h Department of Organismal Biology, Evolutionary Biology Centre, Uppsala University, Norbyvägen 18A, 75236, Uppsala, Sweden; i Department of Veterinary Medicine, University of Perugia, Via San Costanzo 4, 06126, Perugia, Italy

**Keywords:** Apicomplexa, Bacteria, Emerging Infectious Diseases, Fungi, Lizards, Pathogen, Reptilia, Virus

## Abstract

In Europe, reptiles remain among the vertebrates least addressed by conservation actions, despite being significantly impacted by human activities and environmental changes. Pathogenic microorganisms represent an additional yet poorly investigated threat to these animals, largely due to limited veterinary interest, which traditionally prioritises captive species over wild populations. Consequently, comprehensive studies on the pathogens affecting European wild reptiles remain sparse and fragmented, providing limited guidance for conservation strategies or health risk assessments.

This review synthesises the current knowledge on potentially pathogenic microorganisms (namely bacteria, fungi, protozoa *sensu lato* and viruses) in wild, non-marine reptiles across Europe. We analysed 123 peer-reviewed studies from major scientific databases. Results indicate a marked increase in publications over the last two decades, although geographical and research focus biases persist. Southern European countries, notably Spain, Italy and Portugal, dominate the research landscape, while significant gaps exist in Northern and Eastern Europe. Lizards emerge as the most frequently studied hosts, especially in relation to apicomplexan parasites, followed by snakes and turtles. Among microorganisms, protozoa (particularly apicomplexans such as haemogregarines *sensu lato*) are the most frequently documented, whereas bacteria, fungi and viruses are less commonly reported, but significant from conservation and/or zoonotic perspectives. Within the latter, taxa such as *Salmonella*, *Ophidiomyces* and members of the Iridoviridae are relatively well represented. Molecular diagnostics have increasingly supplanted traditional microscopy, yet crucial tools such as culture-based methods and serology remain underutilised, limiting certain aspects of microorganism and disease characterisation.

Bipartite host-microorganism network analysis revealed a specialised, modular structure promoted by specific microbial communities within particular hosts, themselves influenced by potential co-evolutionary dynamics or uneven sampling efforts.

These findings underline the importance of integrating reptile disease ecology into wildlife conservation and public health frameworks, emphasising the urgent need to expand surveillance, particularly in underrepresented taxa and regions, to effectively address emerging disease threats under a One Health approach.

## Highlights

• Reptile pathogens remain neglected, limiting conservation and health measures.
• Protozoans dominate studies; viruses, bacteria, fungi seem underexplored.
• Southern Europe leads reptile disease research.
• Molecular diagnostics surpass microscopy, yet culture and serological methods remain underutilised.
• Host-pathogen interactions are highly specialised, indicating ecological complexity or biased sampling.

## Graphical abstract

**Figure:**
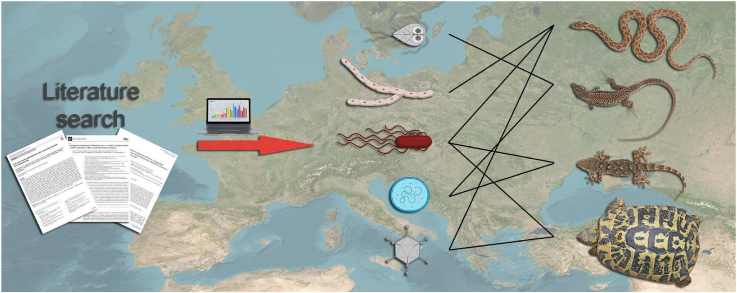

## 1 Introduction

### 1.1 Are pathogenic microorganisms a threat to European reptiles?

Reptiles stand among the least considered and negatively perceived vertebrates in the eyes of the public, presenting a challenge for the conservation efforts directed towards their populations (e.g. Ceríaco, 2012; Janovcová et al., 2019; Pradhan and Yonle, 2022). Moreover, the elusiveness and low detectability of different taxa could complicate field monitoring efforts (Ward et al., 2017; Kolanek and Bury, 2021; Marini et al., 2023). These premises do not favour an order of vertebrates that is experiencing a decline due to the synergistic impact of multiple stressors, including climate change, habitat loss, fragmentation and deterioration, pollution, excessive harvesting, invasive species and intentional persecution (Gibbon et al., 2000; Todd et al., 2010; Böhm et al., 2013; Hoffmann et al., 2010; Falaschi et al., 2019; Cox et al., 2022).

Currently, over 12,000 species of reptiles are recognised worldwide (Uetz et al., 2023). Of these, more than 10,000 have recently been assessed for their threat status, with approximately one-fifth being classified as threatened with extinction (Cox et al., 2022). A similar pattern is observed in Europe, a region that encompasses less than 2 % of the world's reptile species (about 200 species - Speybroeck et al., 2020). In this context, around one-fifth of native species have been identified as being threatened with extinction at the European level, due to factors similar to those reported on a global scale (Cox and Temple, 2009). Without adequate conservation and environmental restoration efforts, this statistic is expected to worsen over time.

Infectious diseases also rank among the threat factors for reptiles, given their susceptibility to a broad range of pathogenic microorganisms (e.g. Schumacher, 2006; Schmidt, 2015; Wirth et al., 2018; Paré and Sigler, 2016; Parrish et al., 2021; Jacobson and Garner, 2021; Marschang et al., 2021; Di Nicola et al., 2022; Schilliger et al., 2023). However, several of these microorganisms are still poorly studied and even for those more thoroughly investigated, their impact on individuals and populations is often unknown. Furthermore, the focus on reptile pathologic conditions is primarily directed towards captive animals, widening the knowledge gap for wild ones (Parrish et al., 2021; Di Nicola et al., 2022; Mendoza-Roldan et al., 2023). In this context, the lack of information is particularly pronounced in Europe, where the limited number of investigations in the wild means that the health of free-ranging reptiles remains a somewhat overlooked condition (Franklinos et al., 2017; Allain and Duffus, 2019; Marini et al., 2023). The relatively low number of scientific articles dedicated to infectious diseases in European wild reptiles and an almost total absence of reviews on the subject constitute evidence of this aspect.

Moreover, some reptile infectious diseases are noteworthy in Europe from a One Health perspective, potentially serving as zoonoses (e.g. Bertrand et al., 2008; Hidalgo-Vila et al., 2008; Traversa et al., 2008; Rinaldi et al., 2012; Díaz et al., 2013; Durand et al., 2013; Ebani, 2017; Reboredo-Fernández et al., 2017; Whiley et al., 2017; Mendoza-Roldan et al., 2019; Mendoza-Roldan et al., 2020; Marin et al., 2013; Mendoza-Roldan et al., 2023). It is notable that, even in this context, many findings concern captive reptiles, for which the European Union member states are currently the largest importers (Engler and Parry-Jones, 2007; Durand et al., 2013; Ebani, 2017).

### 1.2 When is a microorganism considered pathogenic for reptiles?

A pathogen is a biological agent that causes disease in a host (Alberts et al., 2002; Madigan et al., 2019; Meena et al., 2019). Although some larger parasites, such as helminths (see Bruschi, 2022; Thaenkham et al., 2022), are considered pathogens, the term more commonly refers to microorganisms, including bacteria, archaea, fungi, protozoa *sensu lato* (s.l.) and viruses (King, 2012; Archibald et al., 2017; Madigan et al., 2019; Souza Dos Santos et al., 2023). An infection arises when these pathogens invade a host, multiply and interfere with normal cellular and physiological functions. The outcomes of infections vary widely, encompassing asymptomatic colonisation to symptomatic diseases and might involve intricate engagements between microbial virulence factors, host's immune response and microflora interaction (Mandell et al., 2010; Byrd and Segre, 2016). At this stage, a clear distinction should be drawn between primary and opportunistic infections. Some microorganisms can induce infections by invading the tissues and – usually – inducing diseases in healthy hosts due to their intrinsic virulence (Quinn and Quinn, 2011). The latter infectious agents are called primary pathogens because they cause primary infections regardless of the host's immune system. Demonstrating the infectious nature of a primary pathogen involves satisfying Koch's postulates, a criterion of which is “The microorganism must be found in diseased but not healthy individuals” (Koch, 1882). On the other hand, other microorganisms seize the opportunity to cause infection in hosts made susceptible by unbalanced health status, such as impaired immunity, unfavourable environmental conditions or injuries (Quinn and Quinn, 2011). These agents may be called opportunistic pathogens and typically they provoke infections that are the result of complications arising from an underlining affection (i.e. secondary or opportunistic infection). However, the definitions of opportunistic pathogen and infection become more complex when the organism involved is an ectotherm. Reptiles are the unique ectothermic amniotes and their immune response is more dependent and influenced by environmental factors, such as temperature and seasonality, whether compared to endothermic amniotic birds and mammals. For example, reptiles undergo seasonal variation in lymphoid tissues (e.g. thymus and spleen), the efficiency of their immune cell and complement system function is temperature-dependent and they raise their body temperature behaviourally in response to infection (analogous to the fever response in endothermic animals – behavioural fever) (Zimmerman et al., 2010). Moreover, systemic hormonal peaks (e.g. cortisol, testosterone, estrogen) during sensitive seasons as the post-brumation or the mating period usually negatively influence immune activity (Origgi and Tecilla, 2020). Taking into account the host vulnerability required by an opportunistic microorganism to elicit infection and the plasticity of reptilian immune status due to its dependence on the environment, more layers of complexity should be added to the system “reptilian host-microorganism”. Therefore, in reptiles, the term “opportunistic pathogen” possesses more shades of intricacy.

Pathogenic organisms have coevolved with reptiles over millions of years (Mendoza-Roldan et al., 2023). The high biodiversity of microorganisms results in the presence of both nearly taxon-specific entities (e.g. *Scutavirus* spp. – Origgi, 2013) and organisms that are pathogenic across a broad spectrum of hosts (e.g. *Salmonella* spp. *–* Dudek et al., 2019). Some pathogens are considered native to a specific area, while others may be translocated through various pathways, including the trafficking of wildlife (Brianti et al., 2010; Kock et al., 2010). For some of them, the origins or transmission modes are still partially unknown, as in the case of *Ophidiomyces ophidiicola* (Di Nicola et al., 2022; Ladner et al., 2022).

Many of the microorganisms identified in reptiles do not cause clinical disease in these hosts. However, reptiles often act as reservoir hosts, contributing to the sylvatic transmission cycles of some pathogens and/or potentially enabling spillover events. Moreover, their ecological traits, such as ectothermy and frequent exposure to a wide range of vectors and microorganisms, make them valuable sentinel species for monitoring the circulation and emergence of infectious agents, antimicrobial resistance, and chemical residues of relevance to both wildlife and public health.

Since many of the microorganisms affecting reptiles are poorly investigated, as well as the effects they may induce in the host, it is not always straightforward to determine which taxa are pathogenic or not. The Aquatic and the Terrestrial Animal Health Codes by the World Organisation for Animal Health (WOAH) serves as standard and recommendation for the global control of pathogenic agent relevant to animals (WOAH, 2023c,d). In line with that, the Aquatic and the Terrestrial Manual standardise disease diagnosis for species listed in the corresponding Code, streamlining health certification for animals and their products global trade (WOAH, 2023a,b). Nevertheless, unlike other tetrapods and/or amniotes (i.e. amphibians, mammals and birds), reptiles totally lack their class-specific pathogen section and an adequate representation in the global health and diagnostic protocols.

In light of the information presented on the concept of pathogenicity, in this review, we have chosen to include all microorganisms that fulfil, even if only potentially or partially, this notion. Additionally, the work incorporates microorganisms for which knowledge is still insufficient and that cannot be conclusively excluded at this point.

The scarcity of dedicated studies on the pathogens affecting European wild reptiles, as well as the lack of comprehensive information, highlights the need for an overview to assess the current situation. Such data could serve as a starting point to determine the efforts required for future investigations and the measures to be adopted, in terms of wildlife conservation, disease ecology and public health.

The aim of this review is to present the current landscape of microorganisms studied in wild European reptiles, quantifying the focus and research efforts/gaps directed toward different reptilian host taxa and their associated microorganisms. The overarching goal is to quantify and synthesise the existing literature on potentially pathogenic microorganisms in wild European reptiles, identifying which host taxa and pathogen groups have attracted the greatest research attention. By examining temporal and spatial trends in published studies, it aims to evaluate surveillance gaps and underrepresented taxa, regions, microorganism groups and diagnostic methods employed. Using a bipartite host-microorganism network, the study seeks to explore ecological and epidemiological links, giving insights into host specificity and pathogen distribution. Ultimately, this work targets to establish a comprehensive baseline dataset designed to inform future wildlife health surveillance, support pathogen risk assessments and aid in the identification of emerging zoonotic threats, thereby guiding targeted conservation and disease-ecology research across Europe.

## 2 Material and methods

### 2.1 Protocol and eligibility criteria

This systematic review was conducted using the Preferred Reporting Items for Systematic reviews and Meta-Analyses (PRISMA) reporting guidelines (Page et al., 2021). Two of the authors (MRDN and AC) contributed to define the database search strategy.

Peer-reviewed scientific literature, conference papers, posters, abstracts, student theses and books in English that reported data on the detection of (possibly pathogenic) microorganisms in wild European non-marine reptiles were evaluated for inclusion. Review articles or editorials that do not provide new data, studies involving other taxa, experimental studies, articles for which the full-text document was unavailable and studies conducted exclusively in countries other than Europe were excluded. Eligibility criteria are summarised in Table 1.

**Table 1:** Eligibility criteria for article selection.

Criteria	Inclusion	Exclusion
Population	Free-ranging non-marine reptiles	Marine species; captive animals
Microorganisms	Bacteria, fungi, protozoa s.l., viruses	Microorganisms detected in arthropods (e.g. ticks) parasitising reptiles. Helminths, arthropods and other non-microorganism taxa
Range	Expanded geographical definition of Europe according to Speybroeck et al. (2020). See Supplementary Fig. S1	Out of expanded geographical definition of Europe according to Speybroeck et al. (2020)
Interest	Publications reporting on (potentially) pathogenic microorganisms in free-ranging non-marine reptiles	Publications explicitly addressing microorganisms that are not (potentially) pathogenic (e.g. gut microbiota)
Study design	Peer-reviewed scientific literature, conference papers, posters, abstracts, student theses and books	Review articles or editorials that do not provide new data, studies involving other taxa, experimental studies and articles for which the full-text document was unavailable
Language	Full text available in English	Other language than English

### 2.2 Information sources and search strategy

An initial literature search for potentially eligible articles was conducted in March 2023 in the following databases: MEDLINE® via PubMed®, Web of Science Core Collection® and Scopus®. In addition to electronic database searching, other relevant publications were successively checked through Google Scholar® and ResearchGate and, if eligible, opportunistically included. The search strategy was constructed according to JBI guidelines (Pollock et al., 2023) using the PCC search strategy based on the following headings.

- Population: wild non-marine European reptiles
- Concept: Bacteria, viruses, fungi, protozoa s.l.
- Context: (Pathogenic) microorganisms in terrestrial wild reptiles

No date restrictions were applied on the search.

Detailed search strings for each database are provided on **Supplementary Note S1**. The resulting articles were uploaded into Clarivate Endnote Online and duplicates were automatically identified and removed. Additional duplicates were manually eliminated if they had not been detected by the software.

### 2.3 Selection of sources of evidence

Articles underwent two levels of screening independently by two of the authors (MRDN and AC). Title and abstract (level 1) screening was completed based on the above-mentioned exclusion criteria. In case of doubt, articles were not excluded to carry out text screening. Resulting references moved forward to level 2 for full-text screening based on eligibility criteria reported in Table 1.

### 2.4 Data charting process

The eligible articles were read in full and analysed by six of the authors (MRDN, SR, AC, CM, TZ and DM). Data were extracted based on the headings provided in Supplementary Table S1.

Reptile taxonomy was adapted according to Speybroeck et al. (2020) when necessary. Moreover, microbiological data were adapted according to standardised taxonomic following the NCBI Taxonomy database, to ensure consistency and accuracy. For taxonomy that followed serological classification (serogroups and serovars/serotypes), we applied genus-specific rules: for *Leptospira* spp., we followed the guidelines of The Leptospirosis Reference Centre (OIE Reference Laboratory for Leptospirosis - https://leptospira.amsterdamumc.org/leptospira-library/leptospira-strains/, accessed on August 23, 2024) and for *Salmonella* spp., the White Le Minor Kaufmann scheme. Viral families were reported following Marschang et al. (2020) and, when possible, species of viruses were also reported following the Taxonomy Release of the International Committee on Taxonomy of Viruses (https://ictv.global/taxonomy).

### 2.5 Data processing and descriptive statistics

**Fig. 1:**
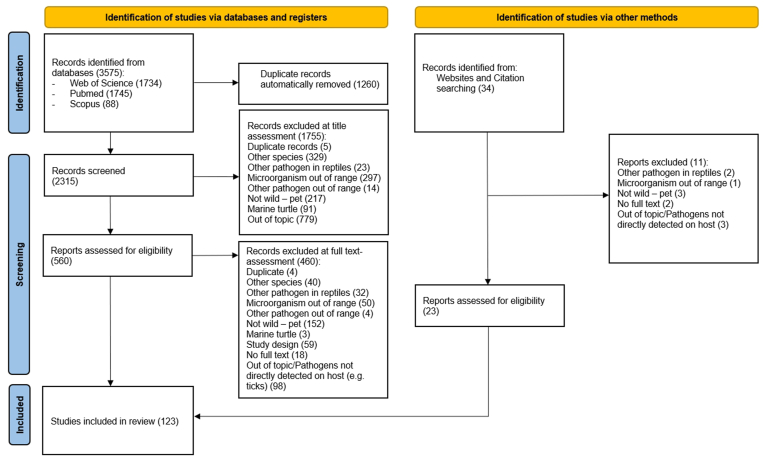
Study selection. Source: PRISMA 2020 flow diagram for new systematic reviews which included searches of databases, registers and other sources (www.prisma-statement.org).

A flowchart was crafted to illustrate the review process, indicating excluded articles and reasons at each level (Fig. 1).

Data for Supplementary Table S1 was extracted from eligible studies. Following manual extraction, the data were standardised under the following headings: ID, Reference, Publication Year (definitive version of the manuscript, taking into account the year of publication of the final volume, if any), States with positive cases, Sampling period, Microorganism taxon/taxa, Microorganism group, Samples type, Microorganism detection/characterisation method, Host taxon/taxa, Host taxon/taxa group, Number of animals sampled in Europe, Number of positive animals/samples, Interaction(s) used for host-microorganism network and Mentions on health conditions. Positive rates were calculated where applicable, following the criteria and methodologies outlined in **Supplementary Note S2**, which also details the exclusion rules.

Descriptive statistics were visualised through graphs generated using Python (v.3.10.2) within a Google Colab environment. The years 2023 and 2024 were excluded from the publications-per-year graph due to their incompleteness, as the literature search concluded before the end of these years and/or because some papers from this period were identified via other methods. A word cloud was created to visually highlight the most frequently occurring terms in the titles of articles identified through the literature search, where word sizes are scaled according to frequency. The word cloud was constructed using WordClouds.com, an online word cloud generator. Moreover, various graphical representations were created to provide an overview of the data derived from the eligible articles identified in the literature review. These include: the number of publications per year; pie charts illustrating the distribution of microorganism groups, host reptile groups and detection/characterisation methods involved (percentages are calculated relative to the total number of categorical occurrences across all studies); heatmaps depicting the relationships between reptile groups and microorganism groups as well as between countries and reptile groups; a map highlighting the frequency of European countries' involvement in the selected studies. It should be noted that when analysing individual microorganism groups (e.g. only bacteria or only viruses), the percentages refer to the subset of studies focusing on that specific group relative to the total of the selected papers: this results in higher relative proportions compared to calculations based on the total number of categorical occurrences across all studies (i.e. pie charts).

### 2.6 Host-microorganism bipartite network and interaction

The host-microorganism bipartite network was constructed based on data obtained from the systematic review of literature on microorganisms detected in wild European reptiles. An interaction was defined as the detection of a microorganism in a reptile individual. Criteria for inclusion/exclusion of articles and calculating the number of interactions to build the network are detailed in **Supplementary Note S3**. The network was represented as a bipartite graph, with reptile genera as nodes on one side and microorganism taxa as nodes on the other. Some microorganism taxa were grouped at family or higher phylogenetic level to enhance clarity and improve clinical relevance (taxa that are definitely pathogenic or zoonotic were left as standalone entries). These aggregated taxa are shown in the simplified version of the bipartite plot and interaction matrix (aggregate version). The criteria used for aggregation in the simplified graphs are detailed in **Supplementary Note S4**. Graphs containing the full taxonomic details (integral version) are available in the **Supplementary files (**Supplementary Figs. S2 and S3**)**.

The structure of host-microorganism interactions was quantified using bipartite networks (Miranda et al., 2013). The interaction network was represented by a matrix consisting of microorganisms as columns, host categories as rows and cell values indicating the percentage of microorganism detection per host specimen. The observed values for network metrics, namely connectance (C), nestedness (N), specialisation (H_2_) and modularity (M), were evaluated according to the definitions provided by Blüthgen et al. (2006), utilising the ‘bipartite’ R package (R Core Team, 2024). A null model comparison was used to assess whether the observed network features outlined host-microorganism “specificity”. The observed values were compared with those derived from 1000 randomised interaction matrices. Randomised matrices maintained the column sums (i.e. the same total number of “infected” subjects for each “pathogen”) as the original matrix (integral matrix not aggregated, see Supplementary Figs. S2 and S3), while allowed for the random redistribution of “infected” specimens across host categories.

## 3 Results

A total of 560 eligible studies were identified through the five database searches. Fig. 1 illustrates the flowchart created to report the number of articles assessed and excluded at each level of analysis, as outlined in methods. A total of 123 articles met the inclusion criteria for this systematic review (the list of 123 included studies is available in Supplementary Table S1, in Appendix I and full references are provided in the reference list).

**Fig. 2:**
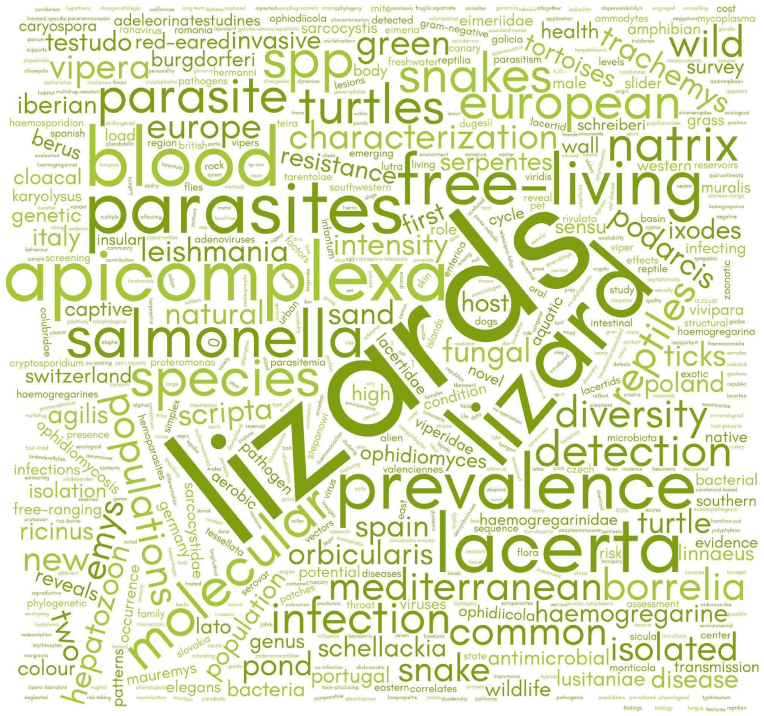
Word cloud generated from the lexical analysis of 123 eligible article titles. Analysis performed using WordClouds.com.

A lexical analysis of 123 article titles using WordClouds.com revealed 1907 tokens (Fig. 2). The frequency distribution highlighted a focus on recurring terms, with lizard(s) (50 occurrences), parasite(s) (31), snake(s) (18), lacerta (17), apicomplexa (16), turtle(s) (16), blood (15), prevalence (14), infection (13), free-living (13), salmonella (12), molecular (11), species (11), european (10), natrix (10) and reptile(s) (10) among the most common.

**Fig. 3:**
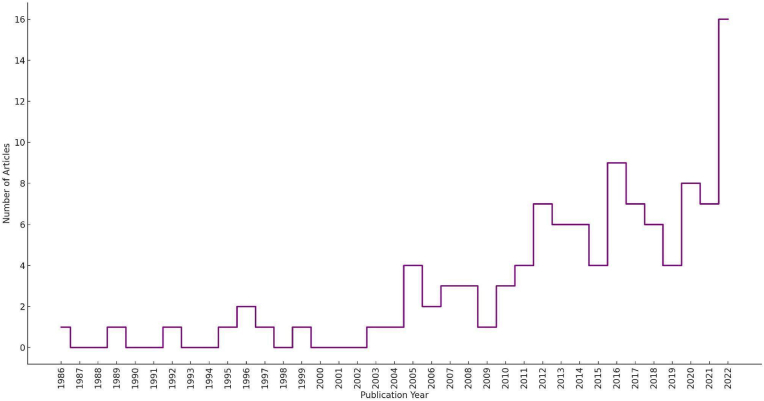
Annual number of scientific publications on microorganisms in European wild reptiles (1986–2022). The data shows a general increase in publications over time, with a sharp peak in 2022. The years 2023 and 2024 were omitted from the graph due to their incomplete data (see Section 2.5).

The number of publications per year investigating microorganisms in European wild reptiles shows a distinct upward trend over the last decades, as reported in Fig. 3. From 1986 to 2000, the number of publications remained consistently low, with no substantial growth observed. However, starting in the early 2000s, there was a gradual increase in the number of studies published annually. This trend accelerated notably after 2010, with a peak recorded in 2022, when the highest number of publications (16) was achieved.

**Fig. 4:**
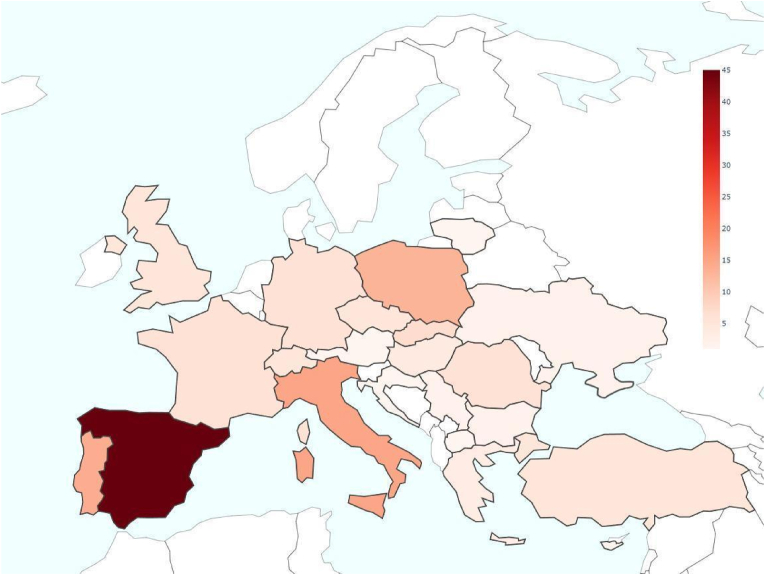
Heatmap illustrating the distribution of scientific publications on pathogen detection in wild reptiles across European countries. The intensity of shading corresponds to the number of publications, with darker colours indicating a higher number of studies.

The heatmap in Fig. 4 illustrates the distribution of scientific publications addressing the detection of microorganisms in wild reptiles across European countries. Each country is shaded according to the number of relevant studies, with darker colours representing a higher number of publications. Spain emerges as the most frequently studied country, with 45 publications, followed by Italy (15), Portugal (14) and Poland (13). Other countries with notable contributions include Slovakia (7), Germany (6), France (6) and Romania (6). The dataset also reveals moderate research activity in the Czech Republic, Switzerland, the United Kingdom and Turkey, each with five publications and slightly lower activity in Hungary (4), Greece (3), Serbia (2), Bulgaria (2) and Ukraine (2). Meanwhile, countries such as Croatia, Lithuania, Cyprus, North Macedonia and Austria each contributed one study. The dataset reveals the absence of studies from some Northern and Continental European countries, as well as from certain Balkan nations.

**Fig. 5:**
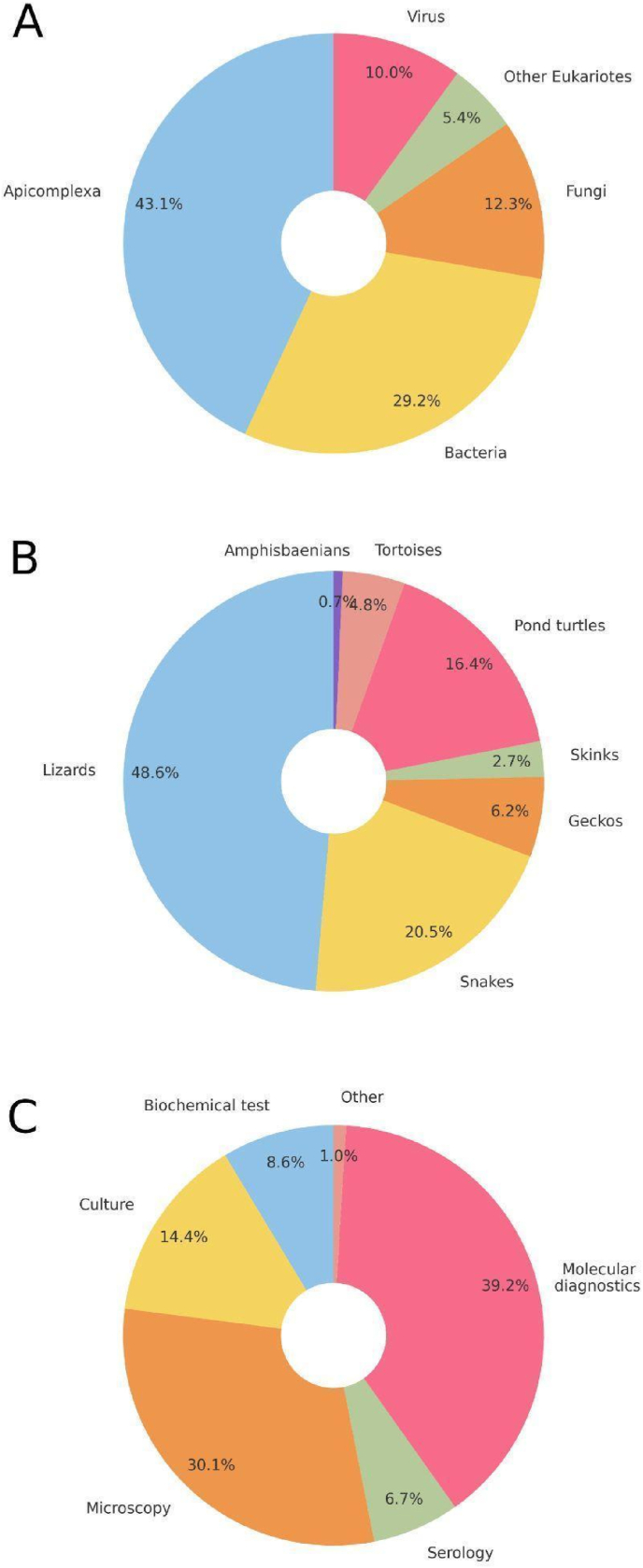
Summary of the diversity of microorganisms, reptilian hosts and diagnostic methods used in studies of European wild reptiles: (A) Proportion of detected pathogen groups (Apicomplexa, bacteria, fungi, viruses and other eukaryotes); (B) Distribution of reptilian hosts by group (e.g. lizards, snakes, pond turtles); (C) Percentage of diagnostic methods used.

In this study, we analysed the diversity of detected microorganisms, their reptilian hosts and the diagnostic techniques used for pathogen detection in European wild reptiles, as summarised in Fig. 5. The most frequently detected group of microorganisms were Apicomplexa (43.1 %), followed by bacteria (29.2 %), fungi (12.3 %) and viruses (10.0 %). A smaller proportion consisted of other eukaryotes (5.4 %) (Fig. 5A). Among reptilian hosts, lizards dominated the dataset, accounting for 48.6 % of total observations (Fig. 5B). Snakes and pond turtles followed at 20.5 % and 16.4 %, respectively, with smaller fractions represented by geckos (6.2 %), tortoises (4.8 %), skinks (2.7 %) and amphisbaenians (0.7 %). Molecular diagnostics were the most commonly employed method (39.2 % - see Fig. 5C). Microscopy, including copromicroscopy and histological techniques, accounted for 30.1 % of methods used. Culture techniques were employed in 14.4 % of cases, while biochemical tests and serology were used in 8.6 % and 6.7 % of cases, respectively. A small percentage (1.0 %) relied on other diagnostic methods.

**Fig. 6:**
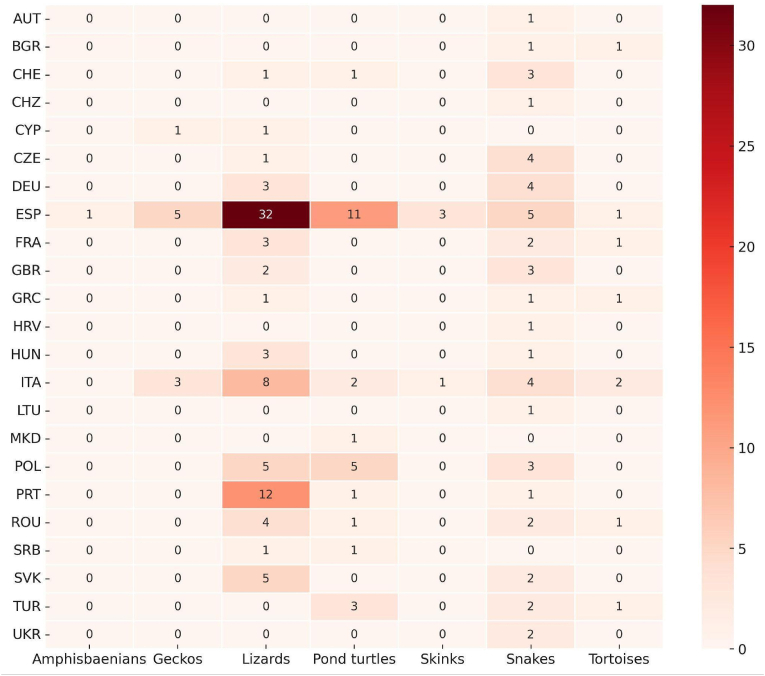
Heatmap displaying the number of studies investigating microorganisms in European reptile groups across different European countries. Each cell represents the count of studies per taxonomic group and country, with the intensity of shading corresponding to the number of records. The data reveal a high concentration of research efforts in lizards, particularly in southern European countries such as Spain, Portugal and Italy, while other regions and taxa remain underexplored.

The heatmap in Fig. 6 illustrates the distribution of studies on microorganisms across reptilian taxa and European countries. The highest concentration of studies pertains to lizards, particularly in Spain (32 studies), followed by Portugal (12 studies) and Italy (8 studies). Conversely, limited or no data is reported for geckos, skinks and tortoises in most countries. Pond turtles have been predominantly studied in Spain (11 studies) and Poland (5 studies). Notably, snakes also show a moderate presence of recorded studies, primarily concentrated in Spain (5 studies), Czechia (4 studies), Germany (4 studies) and Italy (4 studies).

**Fig. 7:**
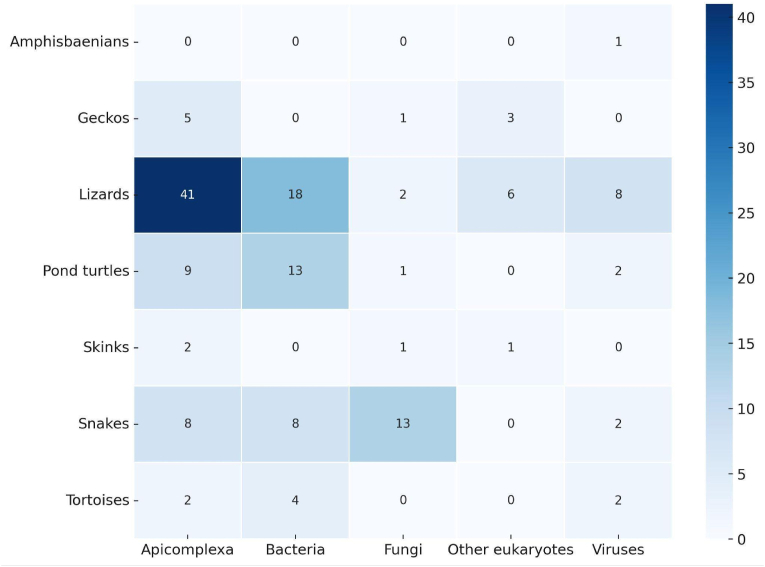
Heatmap illustrating the distribution of studies on microorganism detection across reptile taxa and microbe groups. The values in each cell indicate the number of published studies focusing on specific pathogen groups (Apicomplexa, bacteria, fungi, other eukaryotes and viruses) in different reptile taxa (e.g. lizards, snakes, pond turtles). The numbers reflect study frequencies rather than individual reptile samples. Darker shades indicate a higher number of studies, with lizards showing the highest research focus, particularly for Apicomplexa.

The heatmap presented in Fig. 7 summarises the distribution of published studies focusing on the detection of microorganisms across different reptile taxa. Matrix numbers represent the frequency of publications rather than the number of sampled individuals. Notably, lizards account for the largest share of studies across almost all pathogen groups, with the highest concentration observed in Apicomplexa investigations (41 studies). Chelonians, i.e. pond turtles and tortoises, also exhibit substantial study coverage, particularly in relation to bacteria (13 and 4 studies, respectively). Snakes show a more even distribution of studies among fungi, bacteria and Apicomplexa (8 or more studies for each). Conversely, geckos, skinks and amphisbaenians are underrepresented.

**Fig. 8:**
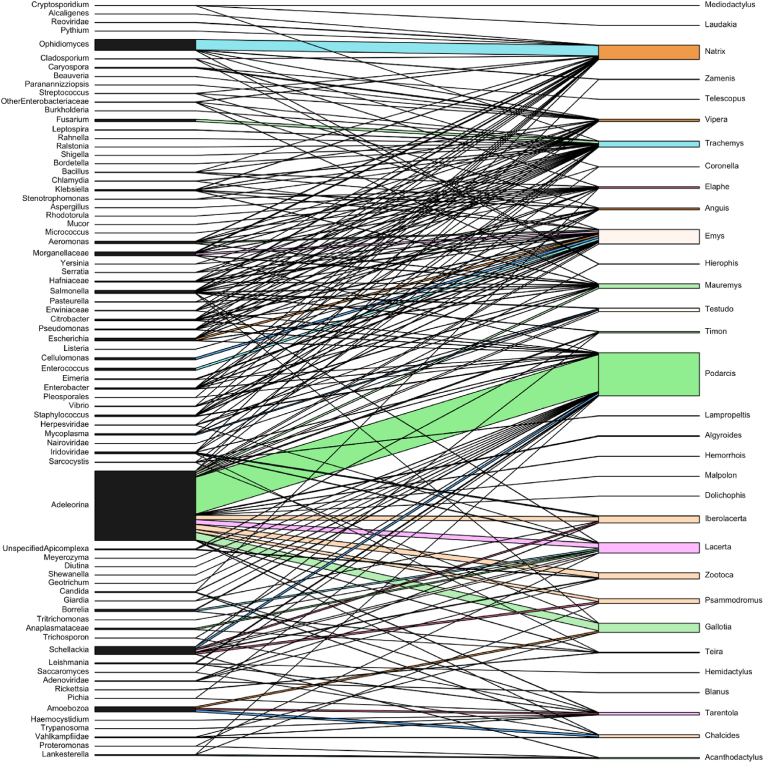
Simplified bipartite plot graph (aggregated version) depicting associations between reptile genera (right) and detected microorganisms (left). Lines indicate specific host-microorganism relationships across diverse reptilian taxa, with line thickness representing the magnitude of interaction between both reptile genus and microorganism taxon.

**Fig. 9:**
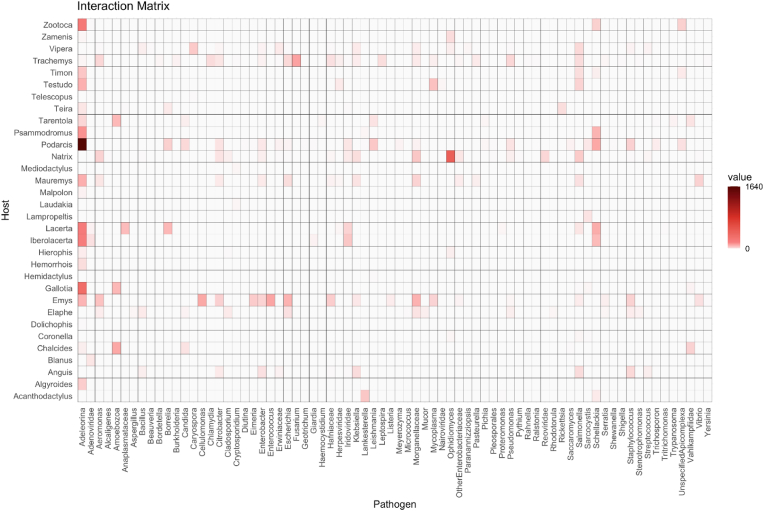
Simplified interaction matrix (aggregated version) displaying the frequency of associations between reptile genera (rows) and potentially pathogenic microorganism groups (columns).

The dataset comprises 6188 total interactions (see the integral version of the dataset in the Supplementary Table S2). Host interactions are notably concentrated, with 51.5 % involving three key taxa: *Podarcis* (31.0 %), *Emys* (10.4 %) and *Natrix* (10.2 %). Interactions resulting from the aggregated version of the matrix (Fig. 8, Fig. 9) and involving *Podarcis* are predominantly with Adeleorina (85 %) and *Schellackia* (4.9 %). *Emys* interacts primarily with *Enterococcus* (15.2 %), *Cellulomonas* (14.5 %), Morganellaceae (11.8 %) and *Escherichia* (11.6 %). Similarly, *Natrix* shows a strong association with *Ophidiomyces* (72.7 %). From the microorganism perspective, in the aggregated version of the matrix the interactions are heavily dominated by Adeleorina (47.7 %) and *Ophidiomyces* (7.8 %), collectively accounting for 55.5 % of all interactions. Adeleorina is most frequently associated with *Podarcis* (55.5 %) and *Gallotia* (11.3 %). Conversely, *Ophidiomyces* interacts almost exclusively with *Natrix* (94.8 %). The structural properties of the observed host-microorganism bipartite network (integral version Figs. S2 and S3) were compared with 1000 randomised networks to evaluate the extent of host-microorganism specificity. This null model analysis included key metrics such as connectance (C), nestedness (N), specialisation (H_2_) and modularity (M) for both the observed and randomised networks, with the results and contrasts summarised in Table 2.

**Table 2:** Structural properties of the observed host-microorganism bipartite network (integral version) versus 1000 random networks.

Metric	Observed	Random Mean ± SD
**Nestedness (N)**	6.168	28.801 ± 0.968
**Specialisation (H_2_)**	4.059	6.866 ± 0.004
**Connectance (C)**	0.087	0.487 ± 0.002
**Modularity (M)**	0.571	0.078 ± 0.002

## 4 Discussion

European reptiles face a variety of threats, ranging from habitat loss and climate change to pollution and direct persecution. Infectious diseases add yet another layer of complexity, particularly for wild populations that have received comparatively little scientific attention. Indeed, much of the existing literature on reptile pathogens focuses on captive individuals, leaving substantial limits in our understanding of how these microorganisms affect free-ranging reptile species in Europe. By systematically reviewing available studies, we aimed to bridge gaps on neglected conditions linked to potentially pathogenic bacteria, viruses, protozoa s.l. and fungi that may influence individual fitness, population health and conservation outcomes. Moreover, several of these microorganisms hold potential significance from a One Health perspective, underscoring the possibility of zoonotic transmission and wider ecological implications. Although the public often perceives reptiles negatively – further hindering support for research and conservation – our findings reinforce the need to view reptile health as a fundamental element to broader ecosystem integrity. The fact that free-ranging reptile populations remain relatively understudied in Europe accentuates the urgency of targeted investigations. Such work is critical to clarifying host-pathogen dynamics, understanding the subtle interplay of opportunistic versus primary infections and promoting effective management strategies that address both environmental pressures and disease risks.

The marked increase of published materials in recent years may be attributed to the growing recognition of reptiles as potential reservoirs of zoonotic pathogens, alongside advancements in molecular diagnostics and pathogen detection techniques (Fig. 3). This trend underscores the increasing awareness of reptile-associated microorganisms in both wildlife conservation and One Health frameworks. Moreover, it highlights the need for sustained and systematic research efforts to bridge existing knowledge gaps and improve our understanding of reptilian pathogen dynamics.

Our findings reveal a significant geographical bias in the current literature, with Southern and Central Europe being more frequently represented, while several Eastern and Northern European countries remain absent from the dataset. In particular Spain, followed by Italy, Portugal and Poland, shows an increasing effort toward the investigation of potentially pathogenic microorganisms in wild reptiles, whereas Scandinavian or some Balkan countries reveal absence of research activities (Fig. 4). This disparity likely reflects differences in research funding, regional herpetofaunal biodiversity and varying levels of scientific interest. Based on the most recent checklist of European herpetofauna by Speybroeck et al. (2020), Europe is home to 200 free-ranging reptile species (excluding sea turtles), comprising 191 squamates and 9 chelonians, distributed across 19 families and 63 genera. Our review reveals that, within this region, more than half of these genera (32 out of 63) and five entire families (i.e. Trionychidae, Chamaeleonidae, Sphaerodactylidae, Typhlopidae and Erycidae) have never undergone any health assessments for potentially pathogenic non-helminthic microorganisms. Surveillance gaps also emerge from a geographical standpoint (Fig. 4). Indeed, entire regions – including Ireland, the Netherlands, Belgium, Denmark, Scandinavia, several Eastern European countries and parts of the Balkans – lack such health studies during the period considered. As a result, the health status regarding these pathogens remains largely unexplored for many taxa. In contrast, countries such as Spain, Italy, Poland and Portugal exhibit the highest number of surveys concerning potentially pathogenic microorganisms. Spain and Italy, in particular, rank among the European nations with the greatest herpetological biodiversity (see Sindaco et al., 2008; Sindaco et al., 2013; Speybroeck et al., 2016), which may partly explain the substantial interest in these types of investigations. However, Greece – despite having a very high number of reptile species too – does not show a comparable level of health screening.

Among the reptile groups most frequently targeted by these studies, lizards emerge distinctly in first place, followed by pond turtles (Fig. 6). Lizards are arguably the most commonly and abundantly encountered reptiles across a wide range of habitats and are generally easier to detect and capture than other reptiles. Similarly, pond turtles are also easily located and captured. Interestingly, snakes rank as the third most frequently investigated group, even though they are challenging to detect and capture. This is probably attributable both to their high species diversity and widespread distribution in certain countries and to the growing interest in emerging fungal diseases affecting them (see below). Regarding the microorganism groups most commonly surveyed, Apicomplexa clearly predominates (Fig. 7). Their frequent occurrence in lizards and the relative ease of detection through microscopic examination of blood smears likely account for this result. Next in prevalence are bacteria, which are widespread and have traditionally been of particular sanitary concern; these are also chiefly reported in lizards, followed by pond turtles. Another noteworthy finding is the substantial number of studies focusing on fungal pathogens, particularly in snakes. The increasing interest in emerging fungal diseases, especially ophidiomycosis, both globally and within Europe (see Di Nicola et al., 2022; Di Nicola et al., 2025), evidently underlies this trend. The data gathered highlight the notable prevalence of protozoan parasites among European reptile populations (Fig. 5A). This predominance may stem not only from their complex life cycles, which frequently involve multiple hosts and their exceptional ecological adaptability, but also from a disproportionate research focus on these particular microorganisms. Regarding host distribution (Fig. 5B), existing studies have heavily emphasised lizards, likely because they are more widely distributed geographically and easier to sample than more elusive or less accessible reptile species. In this context, the observed research bias toward lizards appears closely tied to the substantial volume of work conducted on apicomplexan microorganisms (see Fig. 9).

Molecular diagnostic approaches have come to dominate the field (Fig. 5C), reflecting an ongoing evolution in methodology. In recent years, researchers have increasingly turned to advanced molecular tools, which offer greater precision in identifying microorganisms and conducting genetic analyses. At the same time, traditional microscopy – once a cornerstone of early research – has gradually become less common, demonstrating a clear shift in diagnostic strategies driven by technological progress and the growing demand for detailed molecular data. However, methods such as culture and serology remain underrepresented, limiting efforts to deeply characterise isolates and assess host immune response and exposure to specific microorganisms.

From the 123 selected studies, 62 (∼50 %) focused on eukaryotes microparasites, of which 56 specifically addressed Apicomplexa (∼90 %).

Within the latter phylum, Adeleorina detections were not consistently reported across Europe, with sporadic findings in countries such as Poland, Hungary and Romania, while southern countries, including Spain, Portugal, France, Italy and Greece, highlighted a higher detection rate. This geographic variation likely reflects differences in environmental factors, host species distribution and vector availability. In their natural reptilian hosts, these microparasites are typically incidental findings with minimal clinical relevance (Greiner and Mader, 2006). Veterinary literature suggests that these parasites rarely affect the health of wild or captive reptiles unless exacerbated by stress, age or severe infestations (e.g. Moller and Heatley, 2022; Nardini et al., 2013; Veerselvam et al., 2018). Additionally, *Hepatozoon* infection in lizards (*Podarcis vaucheri*) negatively impacted fitness and locomotor performance, with younger individuals showing higher infection rates, suggesting that lizards tolerate the infection rather than actively combating it (Damas-Moreira et al., 2014). Nevertheless, in aberrant hosts haemogregarine schizonts may induce severe inflammatory lesions in various organs (Campbell, 2006).

The selected studies support a stable evolutionary interaction between host and parasite, with most not addressing clinical conditions, although some explicitly report hosts in good health (see Supplementary Table S1). However, changes in erythrocyte morphology due to gametocytes were observed in *Gallotia* and *Podarcis* lizards by Tomé et al. (2019) and Roca and Galgon (2010), respectively. Similarly, Martín et al. (2007) reported a high parasite load in *Psammodromus algirus* lizards, which was associated with lower immunocompetence and alterations in the chemical signature of femoral gland secretions. Parasite-induced physiological impairments have also been documented: Oppliger et al. (1996) and Walden et al. (2020) both reported impaired mobility and foraging efficiency in *Lacerta vivipara*, linked to decreased haemoglobin levels and an increased proportion of immature erythrocytes, particularly in late summer when parasite loads peak. In contrast, Amo et al. (2004) found that parasitaemia negatively impacted the body condition of Iberian rock lizards (*Lacerta monticola*) in spring. This variability indicates that host-parasite interactions are species- and environment-dependent. Finally, Marzal et al. (2017) reported a significantly higher immune response and lower haematocrit values in Spanish terrapins (*Mauremys leprosa*) with mixed infections compared to uninfected or single-infected individuals. These findings highlight the complex interplay of environmental, host and vector factors in shaping parasite distribution and impact. Although most studies suggest that haemoparasitic Apicomplexa generally have low clinical significance in healthy reptiles, there seems to be limited understanding of the factors influencing pathological outcomes.

Regarding non-haemoparasitic Apicomplexa, *Caryospora* was reported by Mihalca et al. (2003) in three *Vipera* hybrids exhibiting anorexia, weight loss, dehydration and diarrhoea without intestinal lesions (one of these individuals died after four weeks in captivity). *Cryptosporidium* was documented in only two of the selected studies (i.e. in *Trachemys scripta* and *Laudakia stellio*), without mentions of associated pathological signs (Rzeżutka et al., 2020; Schou et al., 2022). The clinical manifestation and outcome of cryptosporidiosis vary considerably across reptile taxa. In lizards and chelonians, *Cryptosporidium* spp. generally show a tropism for the small intestinal mucosa and are often associated with subclinical infections (Eatwell and Richardson, 2019; Walden et al., 2020). In contrast, infection with *C. serpentis* in snakes typically leads to chronic hypertrophic gastritis, which is frequently fatal (Schnellbacher, 2019; Walden et al., 2020). Prey items can act as vehicles for *Cryptosporidium* spp., allowing oocysts to pass through the reptile's gastrointestinal tract without causing infection (Campbell, 2006), a phenomenon known as spurious parasitism. Additionally, reptiles may shed *Cryptosporidium* oocysts that are potentially pathogenic to humans, contributing to zoonotic transmission (Traversa et al., 2008; Mendoza-Roldan et al., 2020). The spillover of non-haemoparasitic Apicomplexa pathogenic to reptiles from captive pets into wild populations could have serious ecological consequences. Therefore, enhanced surveillance is warranted to assess and mitigate potential risks to natural populations.

Out of the 123 selected papers, 62 (ca. 50 %) focused on microparasites, which included 7 (ca. 11 %) on other eukaryotes not belonging to Apicomplexa. All the studies examined reported on healthy animals. Sesma and Ramos (1989) focused on isolating different genera of free-living amoebas from the intestinal contents of reptiles, identifying several genera. Among these, the genus *Entamoeba* represents the only with potential clinical importance, with several species known to be apparently commensals, and others – such as *E. invadens* – to be significant pathogens. The quadrinucleated cyst of *E. invadens* is shed in the faeces, persists in the environment and, when ingested, develops into invasive trophozoites, typically causing necrotic enteritis and hepatitis. Clinical signs include lethargy, depression, hematochezia and death (Wellehan and Walden, 2019). In chelonians, *E. testudines*, *E. barreti* and *E. terrapinae* may cause anorexia, listlessness and watery diarrhoea. Tortoises are more likely to be exposed to temperatures below those optimal for amoeba growth during the winter months, which, combined with food deprivation, reduces the likelihood of encystment. However, once temperatures rise and become favourable for optimal growth, tissue invasion by trophozoites occurs, leading to enterohepatitis. Herbivorous lizards are less susceptible and often serve as carriers. In snakes, the infection manifests as mucoid, bloody faeces. For example, *E. invadens* has been found in water snakes (*Nerodia*), black racers (*Coluber constrictor*) and monitors (*Varanus*) (Walden et al., 2020). Regarding *Leishmania* spp., the 3 studies selected identified *L. tarentolae* and *L. infantum* in *Podarcis* and *Tarentola.* Mendoza-Roldán et al. (2022) reported that 7 out of 227 lizards (3 %) were infected with *L. infantum*, with 1 out of 294 sand flies (*Phlebotomus perniciosus*) captured at the same location also testing positive. Phlebotomine flies, primarily of the genus *Sergentomyia*, along with the transmission of the non-pathogenic species *L. tarentolae*, can also harbour *L. infantum* and *L. major*. Additionally, *L. turanica*, *L. tropica* and *L. donovani*, have been detected in lizards. This raises the hypothesis that lizards and the sand flies associated with them could play a role in the epidemiological cycle of leishmaniasis. Research on vector-borne pathogens is essential to understand the impact of reptile-borne pathogens within the One Health framework (Mendoza-Roldan et al., 2020). Although leishmaniasis is responsible for 20,000 to 40,000 human deaths each year, there has been no published research on the disease in reptiles (Wellehan and Walden, 2019). There is limited knowledge regarding the pathology of *Trypanosoma* spp. in reptiles. It may cause more severe disease in non-endemic hosts that are closely related to their adapted endemic hosts. The diagnosis of trypanosomiasis in reptiles primarily relies on the detection of trypomastigotes in stained blood smears. However, for precise species identification, molecular techniques such as PCR and sequencing are essential (Wellehan and Walden, 2019). Among the studies selected, only Mendoza-Roldan et al. (2022) detected *Trypanosoma* spp. in 3 *Tarentola mauritanica*. Within the selected studies, *Giardia* was identified only in Reboredo-Fernández et al. (2017), in just 5 Iberian lizards. Although disease associated with this organism has not been reported in lizards and their pathological role in reptiles and birds is not as well established, it is recognised as a major enteric pathogen in mammals and remains a significant zoonotic concern (Wellehan and Walden, 2019). In humans, *Giardia* can cause both waterborne and foodborne diarrhoea, along with pathogenic dysbiosis of the intestinal microbiota and vitamin malabsorption, particularly in young children (e.g. in daycare centre outbreaks in developing countries). *G. lamblia* is the most commonly isolated protozoan intestinal parasite worldwide (Leung et al., 2019). Maia et al. (2012) and Kočíková et al. (2018a) found *Proteromonas* spp. in an individual of *Lacerta agilis* and one of *Acanthodactylus erythrurus*, respectively. Phylogenetic studies have revealed that *Proteromonas*, an obligate anaerobic protist commensal in the colon of lizards, is closely related to the genus *Blastocystis*, a group of protozoan parasites of significant medical and veterinary importance. Currently, molecular data is limited primarily to *P. lacertae* (Maia et al., 2012).

A range of fungal genera, from saprophytes to specialised pathogens, have been detected in 16 out of 123 selected papers (13 %). Saprobic molds such as *Cladosporium* are commonly found on reptile skin, but typically as harmless contaminants. For example, Marini et al. (2023) reported *Cladosporium* DNA alongside *Ophidiomyces* in Italian dice snakes, though histology suggested it was a superficial contaminant rather than a primary pathogen (Marini et al., 2023). Nonetheless, *Cladosporium* was also reported to cause mortality events in herptiles (e.g. Grassi et al., 2023). In contrast, members of the *Fusarium solani* species complex are emerging reptile pathogens. These fungi are well-known from “sea turtle egg fusariosis” (STEF) and have been found in eggs of the invasive red-eared slider (*Trachemys scripta*) in Mediterranean habitats (Martínez-Ríos et al., 2022). Martinez-Ríos et al. showed that *T. scripta* eggs naturally harbour *F. falciforme* and *F. keratoplasticum* – pathogens that grow optimally at turtle incubation temperatures – suggesting this alien turtle may serve as a reservoir and vector for STEF fungi (Martínez-Ríos et al., 2022). Among yeasts, surveys of free-ranging lizards have isolated *Candida*, *Trichosporon*, *Meyerozyma* (e.g. *Pichia kudriavzevii*), *Geotrichum* and *Saccharomyces* species from cloacal or faecal samples (Rhimi et al., 2022). For instance, Rhimi and colleagues recovered 364 yeast isolates from Italian lizards, dominated by *Candida albicans* (44 %), *Trichosporon coremiiforme* (12.1 %), *Pichia* (*Meyerozyma*) *kudriavzevii* (8.8 %) and *T. asahii* (7.7 %). Many of these yeasts are opportunistic human pathogens; notably, *C. albicans* and other *Candida* spp., as well as *Fusarium* spp., are included in either the critical or high priority group on the WHO's fungal “priority pathogens” list (WHO, 2022), underscoring their zoonotic and public-health relevance. In fact, reptiles may act as environmental reservoirs: the yeast survey found all isolates were susceptible to antifungals, implying these wild lizards carry pathogenic yeasts without obvious resistance (Rhimi et al., 2022). Besides opportunists, pathogenic fungi with a special tropism for reptiles occur in the Onygenales. *Ophidiomyces ophidiicola*, the cause of ophidiomycosis, has now been documented in Europe (see Table S1 in Di Nicola et al., 2025). This infection, causing skin ulceration and intense mycotic dermatitis, appears to have different degrees of severity in Europe and, among fungi, *Ophidiomyces* is the most represented and studied microorganism (Fig. 8, Fig. 9). The closely related onygenalean *Paranannizziopsis*, causing gross signs overlapping ophidiomycosis, was recently found infecting wild European vipers. Blanvillain et al. (2024b) reported *Paranannizziopsis* in two *Vipera seoanei* specimens from Spain; infections were severe (causing abnormal shedding and emaciation) and one viper likely died of the fungus. Other environmental microbes – such as fungi of various Pleosporales – have occasionally been isolated from reptile skin lesions, though their pathogenic role remains unclear. For example, Klynova and Zinenko (2024) cultured the insect fungus *Beauveria bassiana* from a wild viper's skin lesion; though typically non-pathogenic in vertebrates, *Beauveria* can sporadically infect reptiles. Emerging mycoses may threaten vulnerable herpetofauna, especially as many reports come from regions or species with limited data. Different authors emphasise that their *Ophidiomyces* and *Paranannizziopsis* survey highlights the need to increase sampling efforts in Europe to gauge disease prevalence and impact (Marini et al., 2023; Blanvillain et al., 2024a, 2024b; Di Nicola et al., 2025). The involvement of invasive hosts (e.g. *T. scripta* spreading STEF fungi) and opportunistic saprobes indicates environmental changes or stressors (temperature, humidity, habitat disturbance) may promote fungal emergence. In sum, a variety of fungi – from ubiquitous yeasts (including potential zoonoses) to specialised moulds causing dermatomycoses – are now recognised in European reptiles. Their health impacts range from subclinical carriage to potentially fatal disease and they underscore the gaps in our knowledge of reptile mycobiomes. Continued monitoring (molecular diagnostics, histology) and ecology studies are thus critical to evaluate risks both for reptile conservation and for potential cross-species transmission.

Among the 123 studies, 38 (31 %) focused on Bacteria. In some cases, only healthy animals were sampled (Hacioglu and Tosunoglu, 2014). The majority of the selected studies did not specifically report clinical signs or lesions. Among the few studies that reported clinical signs, the results were mostly unclear. For instance, Mitura et al. (2017), when specifically investigating *Chlamydia* in *Trachemys*, reported a positive specimen with poor body condition and severe conjunctivitis, along with the detection of *Klebsiella* spp. and *Mycobacterium* sp., not clarifying the direct host-pathogen association. Notably, in the study by Hidalgo-Vila et al. (2020), despite the healthy appearance of the red-eared sliders, necropsy revealed hepatic, renal, pulmonary or gastrointestinal abnormalities in the majority of the specimens, but without any association linked to the isolated bacteria. Direct clinical signs with corresponding findings were limited to *Mycoplasma* positivity, as reported by both Ballouard et al. (2022) and Lecis et al. (2011), who observed signs of upper respiratory tract disease (URTD) in *Testudo*. Indeed, *Mycoplasma* sp. is considered a threat for wild tortoise populations and their management (Jacobson et al., 2014).

The most frequently detected group included Gram-negative, often opportunistic and aerobic, bacteria. These microorganisms mainly belong to Enterobacteriaceae, but also include Morganellaceae, Yersiniaceae, Aeromonadaceae and others. Their high detection rate may reflect methodological biases, as these taxa are easily cultured using standard pre-enrichment and selective media commonly employed in diagnostic and research laboratories (routine aerobic culture - see Cushing et al., 2011). While useful for isolating potentially pathogenic strains and enabling phenotypical antimicrobial susceptibility testing, pre-enrichment samples cultivated on and selective media may not reflect the actual composition of the reptilian microbiota. Newer molecular tools, such as NGS-based metabarcoding, allow for a broader and more accurate profiling of microbial communities, including fastidious or unculturable taxa. As mentioned above, despite frequent isolation, no clear clinical associations have been documented between these bacteria and disease in European free-ranging reptiles. However, in captive settings or under stressful environmental conditions, these opportunistic organisms are commonly linked to illness, particularly when immune suppression or suboptimal husbandry is involved. Among the latter group, several zoonotic agents have been identified. In this context, *Salmonella* plays a particularly significant role, which has been found in the absence of clinical signs, constituting a part of the normal reptile microbiota. However, although the role of *Salmonella* as a primary pathogen in reptiles remains partially unclear, reports indicate that snakes are at higher risk of salmonellosis, whereas chelonians exhibit the lowest susceptibility (Pees et al., 2023). In the selected studies, this bacterium was commonly found in the intestinal tract of different species, including Chelonia, Lacertilia and Serpentes and different subspecies of *Salmonella enterica* were detected, including I, II and IIIb. *Salmonella* carriage rates in wild reptiles can be considerable and the risk of zoonotic transmission (i.e. Reptile Associated Salmonellosis - RAS) is substantial. As an example, pet reptiles can carry *Salmonella* at rates over 90 %, contributing to approximately 74,000 reported human cases annually in the U.S., particularly affecting young individuals (11 % of infections among persons aged <21 years - CDC, 2007). RAS typically manifests as self-limiting gastroenteritis, but severe complications such as meningitis, myocarditis, sepsis and even death may occur, particularly in infants, immunocompromised individuals and the elderly (Harris et al., 2010). Clinical signs may vary depending on the serovar and the invasiveness of the strain involved (Pees et al., 2023).

Among other detected bacteria, are worth to mention Gram-positives, including *Listeria*, *Enterococcus*, *Streptococcus* and Coagulase-negative Staphylococci. Some of these microorganisms have a recognised pathogenicity or have emerged as opportunistic/nosocomial pathogens with potential zoonotic implications. Their presence in wild European reptiles, even if detected in 5 of the selected studies, highlights possible reservoirs for environmental contamination and cross-species transmission, posing risks to both animal and human health.

Vector-borne or intracellular pathogens with zoonotic relevance such as *Anaplasma*, *Borrelia*, *Leptospira* and *Rickettsia*, were detected by 11 selected studies. These agents are typically associated with arthropod vectors (e.g. ticks) and/or can circulate between wildlife and humans, posing direct risks to public health and underscoring the importance of including reptiles in surveillance programs for emerging diseases. Their detection in European wild reptiles seems linked to blood or tissue sampling and their presence may signal broader ecological changes, such as shifts in vector distribution linked to climate change or habitat alteration. From a One Health perspective, reptiles could serve as silent reservoirs or incidental hosts, contributing to the maintenance of these pathogens in the environment.

Although microorganisms detected in arthropods parasitising reptiles were not the primary focus of our review, their relevance to vector-borne diseases warrants further consideration. Recent European studies have clearly demonstrated that reptiles can actively participate in the epidemiological cycles of such pathogens. Haematophagous vectors sympatric with reptiles, such as ticks, mites and sandflies, have been shown to carry a wide array of zoonotic agents. For instance, *Podarcis* lizards can host *Rickettsia* spp., facilitating its sylvatic transmission cycles involving *Ixodes ricinus* ticks (Mendoza-Roldan et al., 2021a,b); *Hyalomma aegyptium* ticks collected from endangered *Testudo graeca* tortoises have been found to harbour *Anaplasma phagocytophilum*, *Ehrlichia canis*, and *Coxiella burnetii* (Paştiu et al., 2012); ectoparasitic mites such as *Neotrombicula* spp. may contribute to the maintenance of spotted fever group rickettsiae in reptilian populations (Mendoza-Roldan et al., 2021a,b). Investigating wild reptiles, their ectoparasites, and humans within shared environments represents a key and paradigmatic example of the One Health approach.

Antimicrobial resistance (AMR), including multidrug resistance (MDR), is a growing concern across bacterial and fungal pathogens. While often studied in clinical contexts, AMR is increasingly recognised as an environmental issue. Wild reptiles may serve as sentinels for tracking both bacterial and fungal resistance in natural habitats. Several bacterial genera found in reptiles – such as Enterobacteriaceae, *Enterococcus* spp. and Staphylococci – frequently exhibit resistance to multiple antibiotic classes (e.g. Cushing et al., 2011; Morrison and Rubin, 2020; Cristina et al., 2022; Strompfová et al., 2024). Fungi like *Candida* and *Aspergillus*, occasionally isolated from reptiles, also show resistance traits, raising concerns about emerging mycoses in both human and veterinary settings (Gonçalves et al., 2016; Gow et al., 2022). Reptiles' ecological traits – long lifespan, ground-dwelling habits and exposure to water and soil – make them suitable indicators of AMR spread in transitional zones like wetlands, agricultural borders and peri-urban areas. These environments are often exposed to antimicrobial residues and resistant organisms through runoff or waste. Incorporating reptiles into AMR monitoring frameworks can improve early detection of environmental resistance hotspots. Standardised sampling, broader pathogen screening – including fungi – and integrated ecological data are needed to better understand AMR circulation in wild ecosystems.

From the 123 selected studies, only 13 (10.5 %) included virus detection/isolation. Iridoviridae were detected in lizards, *Natrix* sp., *Anguis* sp. and *Trachemys* sp. in six of these studies (6/13, 46.1 %). Frog Virus 3-like (FV3-like) ranavirus (currently *Ranavirus rana1* – Price et al., 2017), Lizard erythrocytic virus (LEV) (or *Lacerta monticola* ranavirus) closely related to FV3 - (De Matos et al., 2011, De Matos et al., 2013; Stöhr et al., 2015), Common midwife toad virus (CMTV, currently *Ranavirus alytes1* - Borzym et al., 2020) and *Ranavirus* sp. (von Essen et al., 2020) were detected in wild European reptiles. These findings demonstrate the broad host range of ranaviruses and other iridoviruses in European herpetofauna, reflecting their opportunistic nature and their ability to infect ectotherms either primarily or opportunistically. Interestingly, Borzym et al. (2020) was the only study that documented respiratory disease signs in a reptile, *Trachemys scripta*, associated with CMTV (*Ranavirus alytes1*) infection, involving tracheal and splenohepatic pathological alterations. However, in most detections, viruses were isolated from clinically healthy individuals. Indeed, Iridoviruses infections may remain subclinical unless exacerbated by additional stress factors such as brumation, habitat degradation or concurrent infections. Among them, ranaviruses are increasingly recognised as emerging pathogens of conservation concern in reptile populations (Duffus et al., 2015), also for their long environmental persistence (Brunner and Yarber, 2018). Herpesviridae were detected in 3 out 13 studies on viruses (23.1 %). European species of *Testudo* spp. are susceptible to *Testudinid alphaherpesvirus 1* and *3* (currently *Scutavirus testudinidalpha3*). Herpesvirosis in these tortoises, particularly that caused by *Scutavirus testudinidalpha3,* typically presents with conjunctivitis, nasal and oral discharge and respiratory distress, followed by the development of diphtheronecrotic plaques in the oral cavity, tongue and occasionally the oesophagus and intestine, along with neurological signs (reviewed by Origgi, 2013). Ballouard et al. (2022) detected testudinid alphaherpesviruses in 2.7 % of the free-ranging *T. hermanni* tested with PCR; at the same time, of the 28 individuals showing URTD, no one was found with herpesvirus and only 3/28 were detected with *Mycoplasma*. Hidalgo-Vila et al. (2020) detected a herpesvirus sp. in *Trachemys* sp. and *Mauremys* sp., but did not provide sufficient evidence to determine whether the observed pathological lesions were associated to the virus itself or by the presence of multiple bacterial species. Outside of chelonians, a herpesvirus was detected in association with squamous epithelial papillomas in two individuals of *Lacerta viridis* (Literak et al., 2010). Herpesviruses in reptiles are marked by non-specific signs, their latency reduces apparent prevalence, while coinfections can mask herpesvirus as the cause of clinical signs (Okoh et al., 2021). Atadenoviruses were detected in lizards and amphisbaenians in two studies conducted in the United Kingdom and Spain. Although no clinical signs were reported or associated with adenovirus infection, the detection of viral DNA from gastrointestinal swabs suggests a potential for subclinical infections or latent viral circulation within wild reptile populations. Further investigations are needed to assess the pathogenic potential of these viruses under varying conditions and to determine whether they can be associated with clinical disease as seen in captivity (see Wellehan et al., 2004; Ariel, 2011). Only two studies have investigated arboviruses by sampling biological material from European reptiles rather than from arthropod vectors: a novel Orbivirus (family Reoviridae) was accidently detected in blood samples from grass snakes (*Natrix natrix*) in Romania (Tomazatos et al., 2020), while the Crimean-Congo haemorrhagic fever virus (currently *Orthonairovirus haemorrhagiae*) was identified in blood samples from *Testudo* spp. individuals infested with *Hyalomma aegyptium* ticks in Turkey (Kar et al., 2020). These findings highlight the potential role of reptiles not only as passive hosts for tick-borne viruses but also possibly as reservoirs or amplifying hosts for certain arboviruses. Viruses represent an emerging concern, but significant knowledge gaps persist regarding their occurrence and impact in wild European reptiles. The number of viral families investigated remains extremely limited, existing studies are fragmented and many viral detections are incidental, often lacking clear pathological correlations. Moreover, the true effects of viral infections on reptile health, population dynamics and potential zoonotic risks are still largely unexplored. Future research should broaden the spectrum of viral families surveyed, implement longitudinal studies to capture latent and seasonal infection patterns and integrate virological, ecological and pathological data. Given the global relevance of emerging viral diseases and the ecological role of reptiles, such efforts are essential to strengthen both wildlife conservation strategies and One Health surveillance frameworks.

Finally, the observed host-microorganism bipartite network was analysed for four key structural properties: connectance (C), nestedness (N), specialisation (H_2_) and modularity (M). These metrics were compared to those of 1000 randomised networks (Table 2). Both the empirical and randomised networks exhibited low nestedness, with values of 6.168 and 28.801 (±0.968), respectively, indicating an absence of a strongly nested structure. Instead, the empirical network demonstrated significantly higher modularity than the randomised networks (0.571 vs. 0.078 ± 0.002), suggesting that interactions in the observed network are compartmentalised into distinct host-microorganism clusters with strong internal connectivity. The observed network also had notably low connectance (0.087), compared to 0.487 ± 0.002 in the randomised networks. This indicates that only a small proportion of potential host-microorganism interactions were realised, a characteristic commonly associated with specialised ecological interactions, where hosts often harbour unique microbial communities. Similarly, the empirical network exhibited a lower H_2_ value (4.059) relative to the random network mean (6.866 ± 0.004), reflecting a greater degree of specialisation than expected under random conditions. These patterns may result from co-evolutionary dynamics, host specificity or environmental filters that promote the assembly of specific microbial communities within particular hosts. However, it is important to note that these findings could also be influenced by uneven sampling efforts. Researchers may have focused on particular microorganisms (e.g. clinically significant taxa) or specific host taxa (e.g. more detectable species such as lizards), potentially overlooking shared interactions across hosts.

## 5 Conclusions

The present work highlights significant gaps in current knowledge regarding potentially pathogenic microorganisms affecting wild reptiles across Europe. Despite increasing scientific interest, substantial research focus, geographic and methodological biases remain, limiting a comprehensive understanding of reptilian disease ecology. Enhanced efforts should prioritise underrepresented reptile and microorganism taxa and poorly studied regions, particularly in northern and eastern Europe, to provide a more balanced epidemiological picture. Additionally, future investigations should incorporate standardised comprehensive methods including advanced molecular and serological diagnostic techniques as well as histopathology, to accurately assess microorganism diversity, characterise infections and related physiopathology and clarify pathogen-host dynamics in the environment.

Given the demonstrated complexity of reptile-microorganism interactions, as revealed by the network analyses, understanding these ecological relationships is vital for assessing threats to reptile biodiversity, preventing disease outbreaks and managing zoonotic risks within a One Health framework. Bridging these knowledge gaps will enable more informed conservation actions and improve wildlife health surveillance, ultimately benefiting both reptilian conservation and broader ecosystem health. This is particularly urgent considering the escalating pressures from global environmental change, habitat degradation and wildlife trade, which may significantly alter host-microorganism dynamics and lead to new health challenges for wildlife and humans alike.

## CRediT authorship contribution statement

**Matteo Riccardo Di Nicola:** Writing – review & editing, Writing – original draft, Visualization, Validation, Methodology, Investigation, Formal analysis, Data curation, Conceptualization. **Selene Rubiola:** Validation, Investigation, Data curation. **Anna Cerullo:** Writing – review & editing, Writing – original draft, Methodology, Investigation, Data curation, Conceptualization. **Andrea Basciu:** Writing – original draft, Formal analysis. **Claudia Massone:** Writing – review & editing, Writing – original draft, Investigation. **Thomas Zabbia:** Writing – review & editing, Investigation. **Jean Lou Mc Dorne:** Writing – review & editing. **Pier Luigi Acutis:** Writing – review & editing. **Daniele Marini:** Writing – review & editing, Writing – original draft, Visualization, Validation, Methodology, Investigation, Formal analysis, Data curation.

## Declaration of generative AI and AI-assisted technologies in the writing process

During the preparation of this work, the authors used ChatGPT by OpenAI to improve language and readability. After using this tool, the authors reviewed and edited the content as needed and took full responsibility for the content of the publication.

## Funding

Open access funding provided by 10.13039/501100007051Uppsala University.

## Declaration of competing interest

The authors declare that they have no known competing financial interests or personal relationships that could have appeared to influence the work reported in this paper.

## **Appendix I. Li****st of the 123 articles that met the inclusion criteria for this systematic review. Extracted data from each study are provided in Supplementary Table S1**

Allain et al., 2024; Alves De Matos et al., 2011; Amo et al., 2005a; Amo et al., 2005b; Amore et al., 2007; Arizza et al., 2016; Bannert, 1992; Ceylan et al., 2023; De Matos et al., 2013; De Sousa et al., 2012; Dezzutto et al., 2017; Dipineto et al., 2018; Donald et al., 2021; Dormann et al., 2009; Drechsler et al., 2021; Dudek et al., 2016; Ekner et al., 2011; Földvári et al., 2009; Garrido and Pérez-Mellado, 2013; Garrido and Pérez-Mellado, 2013; Guz et al., 2021; Hacioglu et al., 2012; Haklová-Kočíková et al., 2014; Harris et al., 2022; Harris et al., 2012; Herrera-León et al., 2005; Hidalgo-Vila et al., 2007; Horváth et al., 2016; Joudrier et al., 2024a; Joudrier et al., 2024b; Kočíková et al., 2018b; Kopena et al., 2020; Kopena et al., 2021; Krautwald-Junghanns et al., 2013; La Tela et al., 2021; Lazić et al., 2012; Lukac et al., 2017; Maia et al., 2014; Maia et al., 2012; Majlathova et al., 2006; Majláthová et al., 2010; Majláthová et al., 2008; Maričić et al., 2023; Martínez et al., 2011; Matuschka, 1986; Megía-Palma et al., 2018; Megía-Palma et al., 2023; Megía‐Palma et al., 2014; Megía-Palma et al., 2016; Megía-Palma et al., 2022; Meier et al., 2018; Mendoza-Roldan et al., 2022; Mengistu et al., 2022; Merkevičienė et al., 2022; Mihalca et al., 2008; Modrý et al., 1997; Molina-López et al., 2015; Molnár et al., 2013; Musilová et al., 2022; Norte et al., 2015; Nowakiewicz et al., 2015; Origgi et al., 2022; Parejo-Pulido et al., 2023; Pawlak et al., 2020; Přibyl et al., 2023; Rodrigo et al., 2016; Rund et al., 2019; Sagonas et al., 2016; Santana-Hernández et al., 2022; Schnellbacher, 2019; Schmidt et al., 2014; Schönbächler et al., 2022; Schüler et al., 2022; Segade et al., 2006; Široký et al., 2005; Sorci, 1995; Sorci et al., 1996; Storniolo et al., 2022; Szirovicza et al., 2016; Tomé et al., 2021; Tomé et al., 2014; Tomé et al., 2012; Tomé et al., 2018; Tomé et al., 2016; Veith et al., 2023; Volf et al., 1999; Zając et al., 2016; Zechmeisterová et al., 2019.
