# Supplementary material for: Microorganisms in wild European reptiles: bridging gaps in neglected conditions to inform disease ecology research
Source: Int J Parasitol Parasites Wildl. 2025 Jul 5;27:101113. doi: 10.1016/j.ijppaw.2025.101113 (PMC12275881; doi:10.1016/j.ijppaw.2025.101113)
Supplement: Multimedia component 3 [file mmc3.pdf]

Supplementary files

Microorganisms in wild European reptiles: bridging gaps in neglected conditions to inform disease ecology research

Matteo Riccardo Di Nicola[1,2], Selene Rubiola[3,], Anna Cerullo[1] andrea Basciu[4], Claudia Massone[5], Thomas Zabbia[6], Jean Lou MC Dorne[7,], Pier Luigi Acutis[1] and Daniele Marini[8,9,*]

[1] Istituto Zooprofilattico Sperimentale del Piemonte, Liguria e Valle d'Aosta, Via Bologna 148, 10154 Turin, Italy.

[2] Faculty of Veterinary Medicine, Department of Pathobiology, Pharmacology and Zoological Medicine, Wildlife Health Ghent, Ghent University, 9820 Merelbeke, Belgium.

[3] Department of Veterinary Sciences, University of Turin, L.go Braccini 5, 10095 Grugliasco, Turin, Italy.

[4] WAY4WARD, Via Ruggero Bonghi 11/B, 00184 Roma, Italy.

[5] Independent researcher. Via Romana, 06126 Perugia, Italy.

[6] Independent researcher. Via Ungaretti, 22030 Orsenigo, Italy.

[7] Methodological and Scientific support unit, European Food Safety Authority, Via Carlo Magno 1A, 43100 Parma, Italy.

[8] Department of Organismal Biology, Evolutionary Biology Centre, Uppsala University, Norbyvägen 18A, 75236 Uppsala, Sweden.

[9] Department of Veterinary Medicine, University of Perugia, Via San Costanzo 4, 06126 Perugia, Italy.

Correspondence: daniele.marini@ebc.uu.se

Supplementary Note S1

Detailed search strings for each database used for literature searching.

Web of Science

TS=(reptil* OR snake OR herpetofauna OR lizard OR sauria OR squamata OR gecko OR ophidian OR viper OR colubrid OR testudines OR turtle OR tortoise OR slow worm OR chameleon) AND TS=(microorganism OR pathogen OR microbe OR virus OR virosis OR bacteria OR bacterium OR bacteriosis OR protozo* OR protist OR amoeb* OR metamonad* OR Apicomplexa OR mycosis OR fungal OR fungus OR fungi OR fungal disease OR infectious disease OR infection OR mycotoxins)

Scopus

TITLE-ABS-KEY(Europ* OR Albani* OR Andorra OR Armeni* OR Austri* OR Azerbaijan OR Belgi* OR Bosni* OR Herzegovina OR Bulgari* OR Croati* OR Cyprus OR Cyprian OR Czec* OR Denmark OR Danish OR Estonia OR Finland OR Finnish OR France OR French OR Georgi* OR Germa* OR Greece OR Greek OR Hungar* OR Iceland OR Ireland OR Irish OR Italy OR Italian OR Latvia OR Liechtenstein OR Lithuan* OR Luxembourg OR Malta OR Moldova OR Monaco OR Montenegro OR Netherlands OR Dutch OR Macedonia OR Norw* OR Poland OR Polish OR Portug* OR Romani* OR "San Marino" OR Serbia OR Slovak OR Slova* OR Sloveni* OR Spain OR Spanish OR Sweden OR Swedish OR Switzerland OR Swiss OR Turk* OR Ukrain* OR England OR Scotland OR United Kingdom OR "Great Britain" OR British) AND (reptil* OR snake OR herpetofauna OR lizard OR sauria OR squamata OR gecko OR ophidian OR viper OR colubrid OR testudines OR turtle OR tortoise OR slow worm OR chameleon) AND (microorganism OR pathogen OR microbe OR virus OR virosis OR bacteria OR bacterium OR bacteriosis OR protozo* OR protist OR amoeb* OR metamonad* OR Apicomplexa OR mycosis OR fungal OR fungus OR fungi OR “fungal disease” OR “infectious disease” OR infection OR mycotoxins)

PubMed 1

(Europ* OR Albani* OR Andorra OR Armeni* OR Austri* OR Azerbaijan OR Belgi* OR Bosni* OR Herzegovina OR Bulgari* OR Croati* OR Cyprus OR Cyprian OR Czec* OR Denmark OR Danish OR Estonia OR Finland OR Finnish OR France OR French OR Georgi* OR Germa* OR Greece OR Greek OR Hungar* OR Iceland OR Ireland OR Irish OR Italy OR Italian OR Latvia OR Liechtenstein OR Lithuan* OR Luxembourg OR Malta OR Moldova OR Monaco OR Montenegro OR Netherlands OR Dutch OR Macedonia OR Norw* OR Poland OR Polish OR Portug* OR Romani* OR San Marino OR Serbia OR Slovak OR Slova* OR Sloveni* OR Spain OR Spanish OR Sweden OR Swedish OR Switzerland OR Swiss OR Turk* OR Ukrain* OR England OR Scotland OR United Kingdom OR Great Britain OR British) AND (reptil* OR snake OR herpetofauna OR lizard OR sauria OR squamata OR gecko OR ophidian OR viper OR colubrid OR testudines OR turtle OR tortoise OR slow worm OR chameleon) AND (microorganism OR pathogen OR microbe OR virus OR virosis OR bacteria OR bacterium OR bacteriosis OR protozo* OR protist OR amoeb*

OR metamonad* OR Apicomplexa OR mycosis OR fungal OR fungus OR fungi OR fungal disease OR infectious disease OR infection OR mycotoxins)

PubMed 2

(Testudo OR Mauremys OR Emys OR “Trachemys scripta” OR “Trionyx triunguis” OR Laudakia OR Stellagama OR Phrynocephalus OR Trapelus agilis OR Chamaeleo OR Euleptes europaea OR Alsophylax pipiens OR “Hemidactylus turcicus” OR Mediodactylus OR Tenuidactylus OR Tarentola OR Acanthodactylus OR Algyroides OR Anatololacerta OR Archaeolacerta OR Dalmatolacerta OR Darevskia OR Dinarolacerta OR Eremias OR Gallotia OR Hellenolacerta OR Iberolacerta OR Lacerta OR Ophisops OR Parvilacerta OR Phoenicolacerta OR Podarcis OR Psammodromus OR Scelarcis OR Teira OR Timon OR Zootoca OR Ablepharus OR Chalcides OR “Eumeces schneiderii” OR Heremites OR Ophiomorus OR Anguis OR Pseudopus apodus OR Blanus OR “Indotyphlops braminus” OR “Xerotyphlops vermicularis” OR Eryx OR Malpolon OR Coluber OR Natrix OR Coronella OR Dolichophis OR Eirenis OR Elaphe OR Hemorrhois OR Hierophis OR Lampropeltis OR Macroprotodon OR Platyceps OR Telescopus fallax OR Zamenis OR Vipera OR Macrovipera OR Montivipera) AND (microorganism OR pathogen OR microbe OR virus OR virosis OR bacteria OR bacterium OR bacteriosis OR protozo* OR protist OR amoeb* OR metamonad* OR Apicomplexa OR mycosis OR fungal OR fungus OR fungi OR fungal disease OR infectious disease OR infection OR mycotoxins).

Supplementary Note S2

Criteria for built positive rate:

Positive rates were calculated exclusively based on the species resulting on at least one positive sample. Species having negative results were excluded from the calculations.
Positive rates derived from sample sizes below 10 (e.g., 1/5 = 20%) were excluded to ensure reliability. When the number of positive individuals was not explicitly declared, at least one individual was assumed to be positive for inclusion in the positive percentage calculations. When only isolates were reported without clarification of individual-specific relationships, the results were included but considered as separate entries. Studies or results where it was not possible to discern between reptile species or where specific pathogen identification was unclear were excluded from positive percentage calculations.

The criteria for the positive rate calculation, which may vary based on the number of host species, pathogens, sample types, or methods used, are as follows:

1. **Each microorganism by host species**: for each reptile species, the positive rate was calculated as the percentage of positive isolates/detections for a given pathogen relative to the total isolates/detections obtained. For example, in *Emys orbicularis* (total isolates = 49), *Aeromonas hydrophila* was detected in 10/49 isolates (20.4%), *Aeromonas caviae* in 7/49 (14.3%), *Vibrio* sp. in 3/49 (6.1%), and so forth.
2. **Microorganism differentiation within the same host species**: when the same reptile species presented different types of microorganisms or sample origins, such as blood or faeces, results could be divided by microorganism type and sample origin. For example, Haemogregarinae was detected in blood samples with a prevalence of 33/56 (58.9%), while Coccidia in faecal samples had a prevalence of 7/25 (28%).
3. **Same microorganism across different host species**: if a single microorganism was studied, the positive rate was calculated separately for each species. For instance, *Natrix natrix* 15/176 (8.5%), *Natrix tessellata* 44/163 (37%), and *Zamenis longissimus* 11/180 (6.1%).
4. **Mixed approach**: when applicable, microorganism positive rate was determined from multiple tissue samples or sample origins, and percentages were reported separately. For example, *Borrelia lusitaniae* was confirmed in *Lacerta viridis*, where the positive rate was 7/92 (7.6%) from collar scales and 1/47 (2.2%) from toe clips.

Supplementary Note S3

Criteria applied for constructing the host-microorganism network:

1. Taxonomic Criteria and Standardization
    a. The genus level for both reptiles and pathogens was used whenever available. For microorganisms where genus-level identification was not feasible, we employed the lowest possible taxonomic rank.
    b. Data were adapted according to standardized taxonomic following the NCBI Taxonomy database, to ensure consistency and accuracy.
    c. Due to the limited number of viral taxa and lack of consistent genus-level data in some studies, a simplified approach was used for the construction of the host-pathogen network. Viruses were grouped at the family level, as described by Marschang et al. (2020). The used grouping included: Adenoviridae; Herpesviridae; Iridoviridae; Nairoviridae; Reoviridae. The traditional International Committee on Taxonomy of Viruses (ICTV) system was not applied as it did not allow for homogeneous grouping.
    d. Pathogen records with unresolved or recently renewed taxonomic classifications were harmonized to a single genus name (e.g., Mycoplasmopsis spp. were grouped with Mycoplasma species under Mycoplasma genus).
    e. For taxonomy that followed serological classification (serogroups and serovars/serotypes), we applied genus-specific rules: for *Leptospira* spp., we followed the guidelines of The Leptospirosis Reference Centre (OIE Reference Laboratory for Leptospirosis - https://leptospira.amsterdamumc.org/leptospira-library/leptospira-strains/, accessed on 23 November 2023), and for *Salmonella* spp., the White Le Minor Kaufmann scheme.
2. Inclusion and Exclusion of Data
    a. Studies that do not distinguish between reptile species and the isolated pathogen were excluded from the host-pathogen network calculations (e.g. Marin et al. 2013; La Tela et al. 2021).
    b. A paper included in this study reported only microbial isolates without specifying individual-specific relationships. In this case (i.e. Marinez-Rioz et al. 2022), these isolates were incorporated into the bipartite plot without the ability to determine individual-specific associations (likely because eggs were sampled). The actual number of isolates were added to the network based solely on their occurrence because they did not exceed the number of sampled specimens (eggs).
    c. Studies analyzing previously published data already included in the host-pathogen network calculation via other papers were excluded to avoid redundancy (e.g. Tomé et al. 2019 using the same samples from Tomé et al. 2018; Stohr et al. 2015 using a sample from de Matos et al. 2011).
    d. In cases where species identification could not be reliably confirmed as consistent between European and non-European populations, such species were

excluded from the network calculations (e.g. *Acanthodactilus erythrurus* in Megia-Palma et al. 2018).

3. Handling Missing and Ambiguous Data
    a. If the number of positive hosts nor prevalence for a microorganism was not explicitly stated, a single interaction was assumed, representing at least one positive individual linked to the corresponding microorganism (e.g., Martin et al., 2007).
    b. If only the prevalence of positive hosts for a microorganism was stated, the interactions were calculated as percentage of positive on sampled animals (e.g., Storniolo et al., 2022).
    c. If differentiation of the same host and microorganism by sampling location was possible, multiple interactions were recorded based on the number of distinct sites (e.g., Lukac et al., 2017).
    d. When the same microorganism is reported in two species within the same genus, the results are aggregated (multiple interactions), as it is assumed that the individuals are distinct since they belong to different reptile species.
    e. If different values were reported for microbial species from the same genus, when one or more of the genera are not classified at species level (e.g. *Proteus vulgaris* and *Proteus* sp.) on a single host taxon, the higher value was used to represent that microbial genus for the host taxon in the host-pathogen network (e.g. Pawlak et al. 2020).
4. Sample Duplication Avoidance
    a. When multiple microorganisms from the same genus are reported under the same host species, only the highest number of positive outcomes for a specific genus is included in the host-pathogen network (e.g. Sesma et al. 1989; Nowakiewicz et al. 2015; Rhimi et al. 2022). This approach avoids overestimation and ensures conservative reporting, as the presence of co-infections cannot be assumed unless explicitly stated in the study.
    b. In studies where multiple samples were collected from each animal (e.g., from different anatomical sites such as cloacal and oral swabs) and it was not specified which individuals tested positive, only the highest number of positive outcomes from one sample type for a given microorganism was used. This restriction was applied to avoid duplication and overestimation in the host-pathogen interaction network (e.g., in Hacioglu & Tosunoglu, 2014).
    c. Studies employing capture-recapture methods over multiple years were carefully scrutinized to avoid counting the same individual multiple times. When discrepancies arose, the highest plausible number of positive cases was included in the network (e.g., Sorci et al. 1996).
5. Aggregation of Results
    a. When the same microorganism was reported in two species within the same genus, the results were aggregated (multiple interactions), as it is assumed that the individuals are distinct since they belong to different reptile species.

Supplementary Note S4

Aggregation criteria for Host-Pathogen Network Visualization. To enhance the readability of the host-pathogen bipartite interaction plot and heatmaps, as well as enhance clinical relevance (definitively pathogenic or zoonotic taxa were left as standalone entries), we applied taxonomic aggregation. The aggregations used in the simplified illustrations (**Fig. 8, 9**) were applied to ensure that taxa represented reflected meaningful and cohesive biological relationships while avoiding excessive fragmentation due to closely related taxa. This approach allows for clearer visualization of interaction patterns and simplifies the interpretation of the network structure.

The following specific groupings were established:

1. **Adeleorina:**
   Included genera: *Haemogregarinidae, Haemogregarina, Karyolysus, Hemolivia, Hepatozoon,* and *Adeleorinae.*
2. **Amoebozoa:**
   Included genera: *Acanthamoeba, Stenamoeba, Pessonella, Vannella, Echinamoeba, Hartmannella, Platyamoeba,* and *Filamoeba.*
3. **Erwiniaceae:**
   Included genera: *Pantoea* and *Erwinia.*
4. **Morganellaceae:**
   Included genera: *Morganella, Providencia,* and *Proteus.*
5. **Hafniaceae:**
   Included genera: *Edwardsiella* and *Hafnia.*
6. **Other Enterobacteriaceae:**
   Included genera: *Plesiomonas, Raoultella, Yokenella, Leclercia,* and *Lelliottia.*
7. **Vahlkampfiidae:**
   Included genera: *Naegleria, Vahlkampfia,* and *Adelphamoeba.*
8. **Pleosporales:**
   Included taxa: Pleosporales and *Alternaria.*
9. **Anaplasmataceae:**
   Included taxa: *Anaplasma,* Anaplasmataceae, and *Cryptoplasma.*

Supplementary Figure S1.

Map showing the Expanded geographical definition of Europe according to Speybroeck et al. (2020). Map of Europe generated using QGIS 3.28. Satellite data were sourced from the ESRI Satellite file.

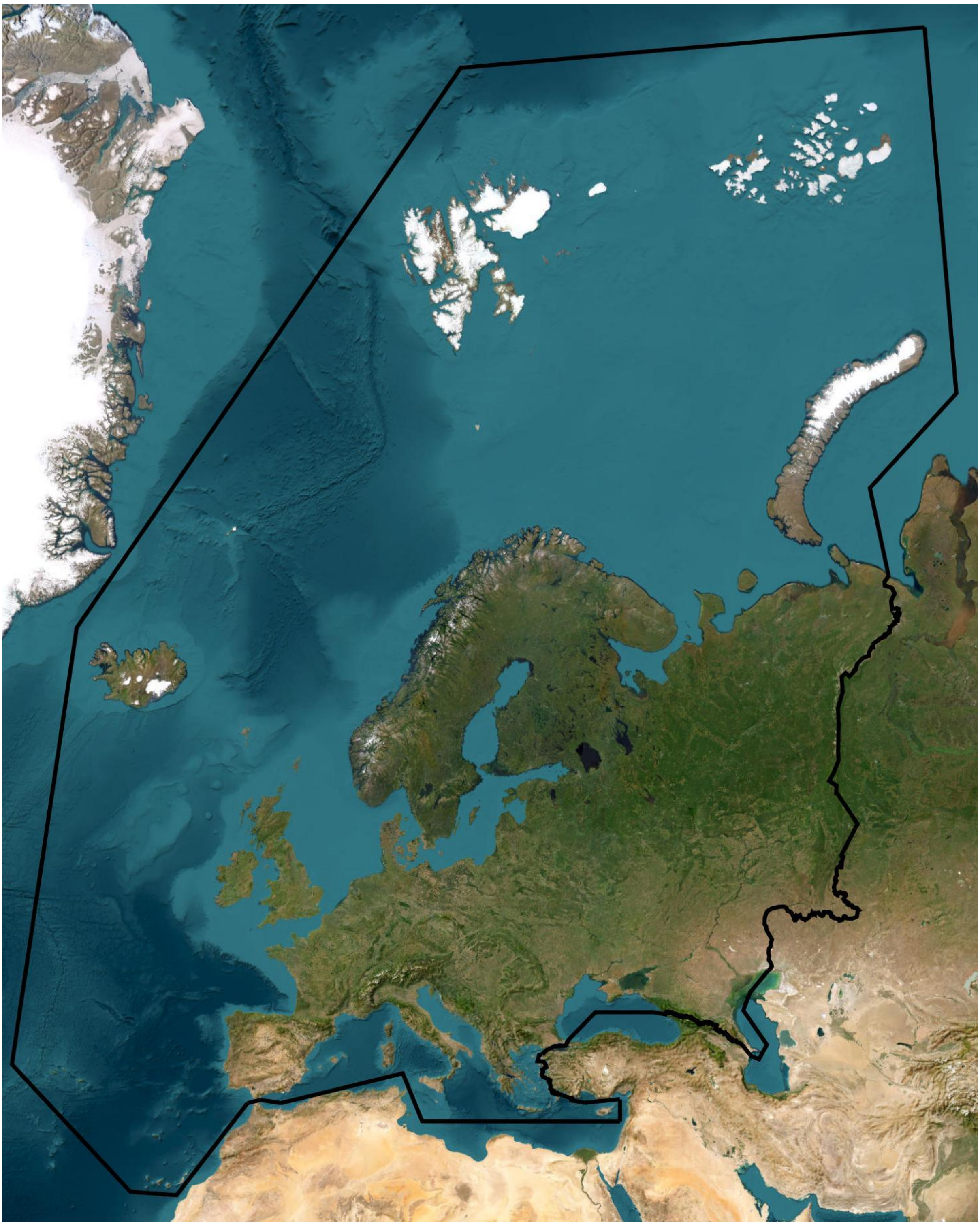

Supplementary Figure S2

Bipartite plot (integral version) graph depicting associations between reptile genera (right) and hosted microorganisms (left). Lines indicate specific host-microorganism relationships across diverse reptilian taxa, with line thickness representing the magnitude of interaction between both reptile genus and microorganism taxon.

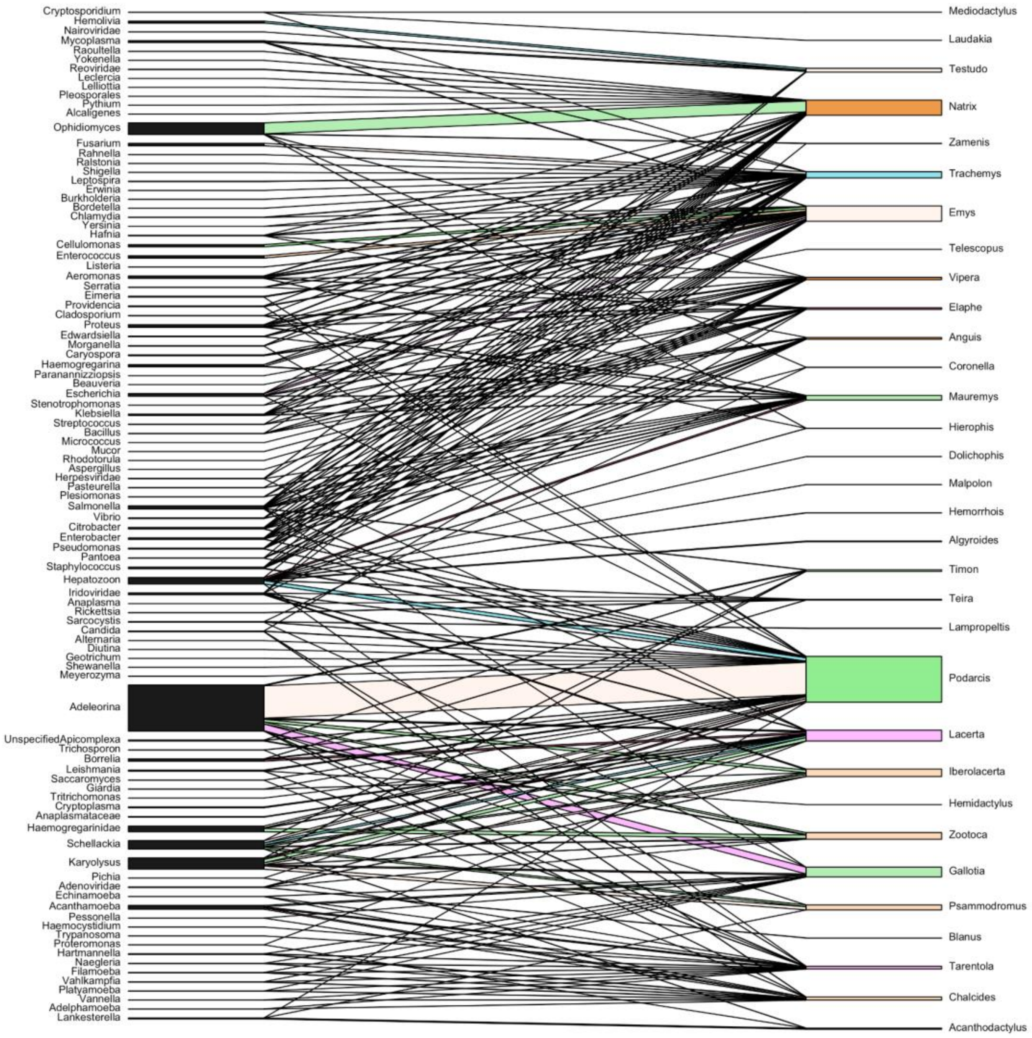

Supplementary Figure S3

Interaction matrix (integral version) displaying the frequency of associations between reptile genera and microorganism groups.

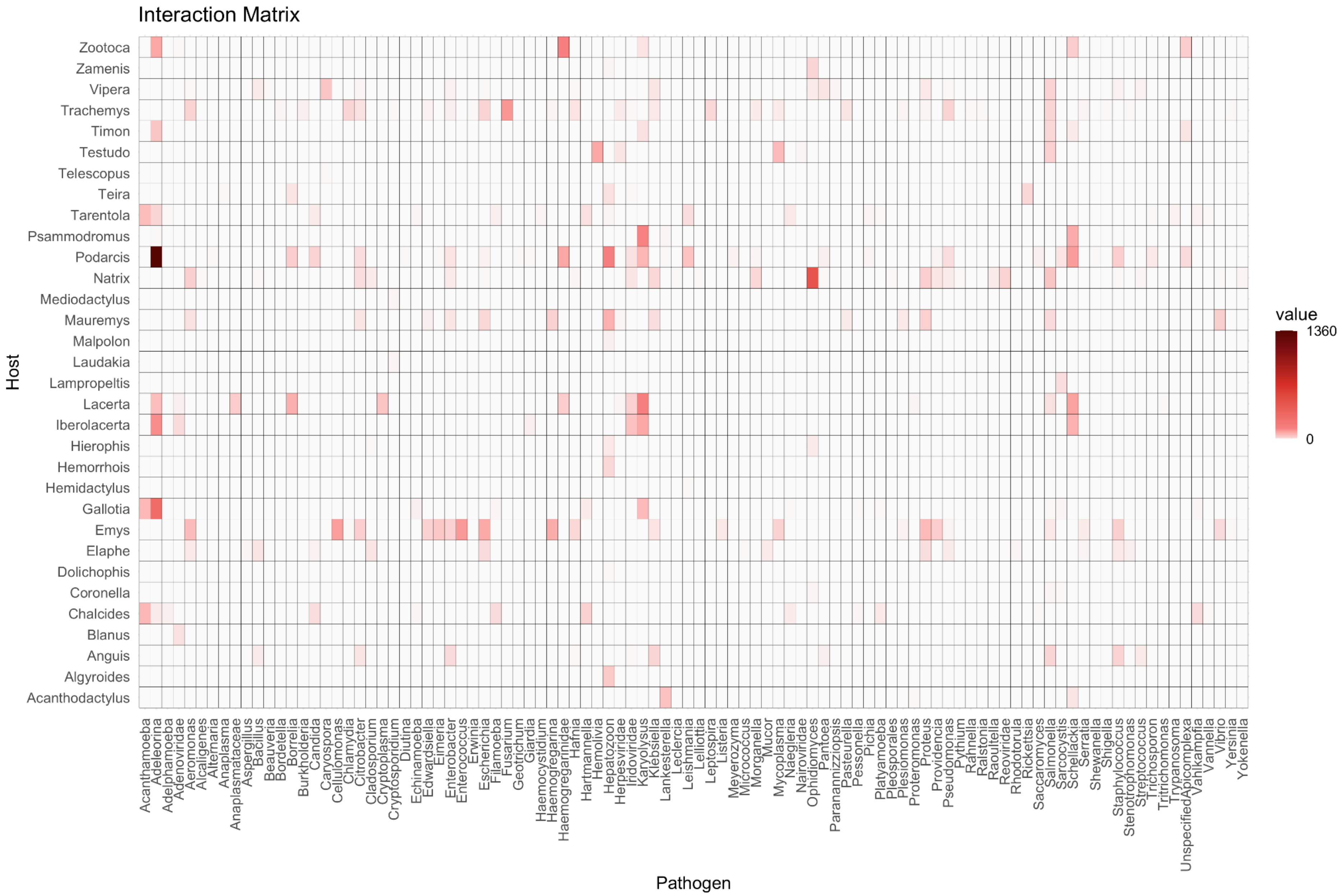

Interaction Matrix
Host
Pathogen
value
1360
0
Zootoca
Zamenis
Vipera
Trachemys
Timon
Testudo
Telescopus
Teira
Tarentola
Psammodromus
Podarcis
Natrix
Mediodactylus
Mauremys
Malpolon
Laudakia
Lampropeltis
Lacerta
Iberolacerta
Hierophis
Hemorrhois
Hemidactylus
Gallotia
Emys
Elaphe
Dolichophis
Coronella
Chalcides
Blanus
Anguis
Algyroides
Acanthodactylus
